# XIST-induced silencing of flanking genes is achieved by additive action of repeat a monomers in human somatic cells

**DOI:** 10.1186/1756-8935-6-23

**Published:** 2013-08-01

**Authors:** Jakub Minks, Sarah EL Baldry, Christine Yang, Allison M Cotton, Carolyn J Brown

**Affiliations:** 1 Department of Medical Genetics, Molecular Epigenetics Group, University of British Columbia, Life Sciences Institute, 2350 Health Sciences Mall, Vancouver, BC V6T 1Z3, Canada

**Keywords:** X-chromosome inactivation, XIST, Long non-coding RNA, Eutherian dosage compensation, Gene silencing

## Abstract

**Background:**

The establishment of facultative heterochromatin by X-chromosome inactivation requires the long non-coding RNA XIST/Xist. However, the molecular mechanism by which the RNA achieves chromosome-wide gene silencing remains unknown. Mouse *Xist* has been shown to have redundant domains for *cis*-localization, and requires a series of well-conserved tandem ‘A’ repeats for silencing. We previously described a human inducible *XIST* transgene that is capable of *cis*-localization and suppressing a downstream reporter gene in somatic cells, and have now leveraged these cells to dissect the sequences critical for *XIST*-dependent gene silencing in humans.

**Results:**

We demonstrated that expression of the inducible full-length *XIST* cDNA was able to suppress expression of two nearby reporter genes as well as endogenous genes up to 3 MB from the integration site. An inducible construct containing the repeat A region of *XIST* alone could silence the flanking reporter genes but not the more distal endogenous genes. Reporter gene silencing could also be accomplished by a synthetic construct consisting of nine copies of a consensus repeat A sequence, consistent with previous studies in mice. Progressively shorter constructs showed a linear relationship between the repeat number and the silencing capacity of the RNA. Constructs containing only two repeat A units were still able to partially silence the reporter genes and could thus be used for site-directed mutagenesis to demonstrate that sequences within the two palindromic cores of the repeat are essential for silencing, and that it is likely the first palindrome sequence folds to form a hairpin, consistent with compensatory mutations observed in eutherian sequences.

**Conclusions:**

Silencing of adjacent reporter genes can be effected by as little as 94 bp of XIST, including two ‘monomers’ of the A repeat. This region includes a pair of essential palindromic sequences that are evolutionarily well-conserved and the first of these is likely to form an intra-repeat hairpin structure. Additional sequences are required for the spread of silencing to endogenous genes on the chromosome.

## Background

To ensure dosage compensation of X-linked genes between males and females, eutherian females silence one X chromosome [1]. The minimal region required for X-chromosome inactivation contains the non-coding (nc) RNA gene *XIST*, which is expressed solely from the inactive X chromosome [2]. Experiments in mice have shown that *Xist* is both required and sufficient for inactivation; however, the mechanism by which the XIST/Xist RNA causes chromosome-wide gene silencing remains elusive (reviewed in [3]). XIST localizes *in cis* to the chromatin of the inactive X chromosome [4], suggesting a potential role in targeting silencing complexes to the chromosome. The alternatively spliced and polyadenylated RNA is over 15 kb long in all eutheria examined. Overall the gene is only weakly conserved among mammals, but its regions of repetitive sequences called repeat A to F show better conservation [4,5]. Additionally, exon 4 of *XIST/Xist* is well-conserved, and shows homology with the protein-coding *Lnx3* gene, from which the *Xist* gene may have evolved by the addition of sequences from transposable elements [6,7]. Intriguingly, in marsupials, *Lnx3* remains protein-coding and *Rsx3* encodes an RNA that is similar to *XIST* in that the long non-coding, repeat-rich RNA is transcribed from and associates with the inactive X chromosome [8]. While there is no sequence conservation between *Rsx3* and *XIST*, both are able to silence *in cis*, and show regions of putative stem-loop structure, supporting the idea that these long ncRNAs may be serving as adapter molecules containing different protein-recognition motifs to recruit components of the gene-silencing machinery to the inactive X chromosome.

As X-chromosome inactivation is a developmental process, most studies of *Xist* function have been undertaken in mice, where embryonic stem (ES) cells or embryos can be analyzed during the inactivation process. Human ES cells have demonstrated considerable epigenetic instability (for example, [9]) and studies of human embryos are necessarily restricted ([10,11]). However, the potential differences in the inactivation process between mice and humans, suggested by both differences in the regulation of the *XIST* gene and the number of genes escaping inactivation (reviewed in [12]), led us to develop an inducible model to study human *XIST* action [13]. Induced expression of *XIST* in the immortal HT1080 fibrosarcoma cell line is able to induce some features of an inactive X, including XIST localization, silencing of a co-integrated reporter gene, depletion of repetitive (CoT1) RNA, and the acquisition of some heterochromatic histone modifications associated with the inactive X.

Previous studies in mice targeted a panel of truncated inducible *Xist* transgenes to the single X chromosome in a male ES cell and demonstrated that redundant sequences were involved in localization of the mouse Xist RNA to the chromosome [14], with a construct containing only approximately 3 kb of *Xist* cDNA, including the well-conserved A repeat region, able to localize to and repress the single X chromosome. Furthermore, chromosomal silencing was fully compromised when the 5’ region encompassing repeat A was deleted [14], but concatamers of a synthetic version of these repeats were able to replace the A repeat region. A near normal complement of 7.5 repeats or an increase to 12 repeats fully recapitulated silencing, while 5.5 repeats showed less silencing and 4 repeats were only minimally active [14]. Therefore, in mice the A repeats are necessary for silencing, but additional redundant domains of *Xist* are involved in localization to the chromosome and the presence of different domains supports models that the RNA is serving as an adaptor to bring different epigenetic silencing proteins to the inactive X.

A number of chromatin remodeling proteins associate with the inactive X chromosome resulting in the acquisition of many histone modifications characteristic of heterochromatin (reviewed in [3]). The binding of many of these proteins is *Xist*-dependent; and it has been shown that the A repeat region interacts *in vitro* and *in vivo* with components of PRC2 [15-17]. Surprisingly, however, a silencing defective Xist RNA that lacks the repeat A region is still able to recruit PRC2, PRC1, SAF-A, ASH2L and macroH2A1 to the inactive X chromosome in ES cells (reviewed in [3]). In contrast, a similar deletion in transgenic mice failed to produce Xist RNA, suggesting an important regulatory role for the repeat A region [18]. Furthermore, interaction with the transcriptional repressor YY1 [19] has been shown to occur at the mouse C repeat region and while a direct interaction with the A repeat region has been reported for the splicing factor ASF/SF2, this has been proposed to have a role in enabling proper processing of the Xist RNA facilitating choice of the future inactive X chromosome [20]. Therefore, despite the growing body of literature on XIST/Xist-interacting partners and identification of a critical role for the A repeat region, understanding how *XIST/Xist* expression leads to gene silencing remains elusive. Contributing to the challenge is the large size of the XIST RNA, and that the monitoring of silencing at distal sites requires both silencing and spread of the RNA along the chromosome.

The palindromic nature of the repeat A core sequences suggests their involvement in forming a distinct secondary RNA structure, and several alternative but mutually exclusive structures have been suggested. The first model proposed that each of the two palindromes forms a hairpin and thus the repeat A region of XIST RNA folds into a two-hairpin 8- or 9-mer [14]. This structure was supported by the abrogation of silencing activity in a construct with two base alterations that would disrupt the putative first hairpin. However, an *in vitro* analysis of repeat A structure by fluorescence resonance energy transfer, as well as sensitivity to RNases that specifically digest single- or double-stranded RNA regions, proposed an alternative structure. The first palindrome was suggested to engage in pairing between two separate monomers, rather than within repeat A monomers, and the model proposed that the second palindrome did not form a defined structure [16]. Recently, a third option, supported by nuclear magnetic resonance analyses of repeat A monomer and dimer structures, suggested that under *in vitro* conditions, the first palindrome forms a hairpin, while the second palindrome engages in pairing between repeat A units [21,22].

Our previously reported inducible transgenic system in the immortal fibrosarcoma line HT1080 provides a tractable system to study the RNA sequences involved in gene repression by XIST [13]. Here, we focus on refining the minimal *XIST* sequence necessary for *cis*-regulated silencing, independent of the developmental signals that establish mono-allelic *XIST* expression in females. We demonstrate silencing of reporter genes by expression of less than 100 bp of *XIST* containing two consensus repeat A monomers.

## Results and discussion

### Repeat A is sufficient for *XIST*-dependent reporter gene silencing

**Figure 1:**
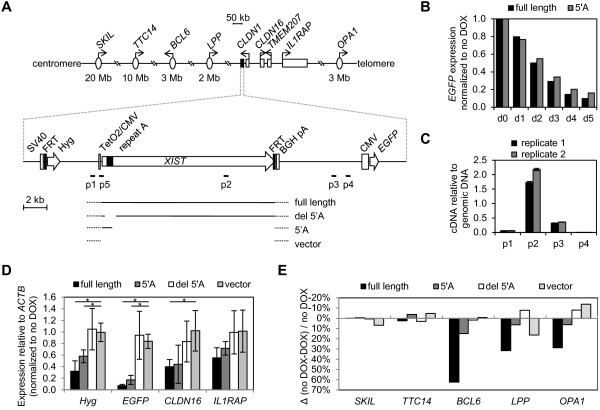
**The repeat A region of XIST is necessary and sufficient for silencing of flanking reporter genes. (A)** Approximate location of genes analyzed on chromosome 3 relative to the schematic of full-length *XIST*cDNA construct showing regions included in shorter *XIST* constructs and location of qRT-PCR primer pairs p1 to p4 and p5 (vector primer pair used to amplify all *XIST* constructs). **(B)** Enhanced Green Fluorescent Protein gene *(EGFP)* expression following one to five days (d1 to d5) induction of full-length *XIST* or 5’A, measured by flow cytometry and shown relative to d0. **(C)** qRT-PCR analysis of expression within full length *XIST* transgene (p2) and upstream (p1) and downstream (p3, p4) of *XIST* sequence. Genomic DNA was used to normalize for amplification efficiency. Location of qPCR amplicon positions is shown in Figure 1A. **(D)** Expression of the reporter genes (Hygromycin gene *(Hyg)* and *EGFP*) and endogenous genes *CLDN16* and *IL1RAP* following five days of transgene induction measured by qRT-PCR, relative to expression in uninduced cells (d0) and normalized to *ACTB* expression. Transgene constructs were full XIST, 5’A only, full XIST lacking the 5’A region or vector with no XIST as indicated. Error bars indicate ± 1 S.D. of four to six biological replicates. Significance (*P*-value <0.05) was calculated using a Mann–Whitney test comparing each transgene construct with the vector alone construct. **(E)** Allele-specific silencing of flanking endogenous genes following five days of transgene induction. The percent change in allelic ratio upon DOX induction relative to the ratio without DOX was measured by pyrosequencing for expressed polymorphisms in five genes up to 20 Mb from the integration site (see A). Transgene constructs were full XIST, 5’A only, full XIST lacking the 5’A region or vector with no XIST as indicated. Two technical replicates of three biological replicates were averaged for each datapoint.

We have previously shown that an inducible transgenic *XIST* is capable of silencing an Enhanced Green Fluorescent Protein gene *(EGFP)* reporter in human somatic cells, while a construct lacking the repeat A region failed to silence the *EGFP* gene [13]. Similarly, inducible mouse constructs have been shown to require the repeat A region for silencing of the X chromosome in mouse ES cells [14]. The RNA induced from the full-length *XIST* cDNA construct localizes to the autosome upon which it has integrated [13]; however, the *EGFP* reporter construct is located only 7.7 kb 3’ of *XIST* in HT1080 male fibrosarcoma cells (see Figure 1A) and, thus, may not require the localization domains of XIST for silencing. Therefore, to test whether the repeat A is sufficient for proximal gene silencing, we induced expression of a construct containing only repeat A sequence (5’A) and measured *EGFP* expression by flow cytometry (Figure 1B). The extent and dynamics of *EGFP* silencing by repeat A mimicked that of the full *XIST* construct over five days following induction of the construct’s expression by doxycycline (DOX), suggesting that the ability of *XIST* to silence the proximal *EGFP* reporter gene is attributable to the repeat A region.

To confirm that silencing results from an XIST RNA-related, sequence-specific effect rather than transcriptional interference, we demonstrated that transcription of the DOX-induced *XIST* transgenes ceased before the reporter construct. While some transcripts were present downstream of the polyadenylation site, transcription was completely absent at a site approximately 2 kb 5’ of the *EGFP* promoter (Figure 1C). Our conclusion that silencing is not due to transcriptional interference is further supported by XIST-dependent attenuation of the expression of the hygromycin resistance gene (*Hyg*) located upstream of *XIST* and absence of gene silencing with vector lacking *XIST* sequences (Figure 1D).

### Endogenous gene silencing induced by full-length XIST

In order to explore whether XIST RNA is able to induce silencing of the endogenous genes flanking the integration site, we identified the FRT integration site into which subsequent single-copy integrations were directed. DNA-FISH using the *XIST* cDNA identified the full-length transgene as integrated onto the der(11)t(3;11) of 46,XY,del(1)(p21),i(3)(p10),i(3)(q10),der(4)t(1;4)(p21;p16),der(5)t(5;5)(p15;?),der(11)t(3;11)(q11;q25) cells. We used inverse PCR from primers in the pFRT/lacZeo plasmid to identify the 3q FRT integration site as just downstream of the *CLDN1* gene (Figure 1A). Low expression levels of the *CLDN1, TMEM207* and *LEPREL1* genes prevented a reliable analysis of these adjacent genes by qRT-PCR. Using qRT-PCR following induction of full-length *XIST,* we observed significant silencing of *CLDN16*, a gene located approximately 100 kb downstream of *XIST* (Figure 1D). Neither the construct consisting only of repeat A, nor the construct containing a deletion of repeat A showed significant silencing of *CLDN16* upon induction, although there was a non-significant reduction with the repeat A-containing construct. *IL1RAP*, which is located a further 120 kb downstream (that is, 220 kb from *XIST*) did not show significant *XIST*-induced silencing, although there was a non-significant drop in expression. The decrease in *CLDN16* transcription is consistent with the almost complete silencing of the *cis*-located allele; however, attempts to confirm silencing of the XIST-associated allele by FISH failed, presumably due to the relatively low expression levels of *CLDN16*. In order to examine whether one allele of endogenous genes was being silenced, we identified more distal genes that contained an expressed polymorphism, and thus provided an opportunity to probe allelic silencing. At the DNA level these genes show an allelic ratio of approximately 66%, consistent with the presence of a single allele on the der(11)t(3;11) and the alternate allele in two copies on the isochromosome 3q. Upon DOX treatment there was a significant decrease in relative expression of the single allele for *BCL6*, *LPP* and *OPA1* (Figure 1D), shown as a change upon DOX induction, relative to the expression in cells without DOX treatment, as there can be variations in allelic expression levels. Similar to the q-PCR results with *CLDN16*, constructs containing *XIST* lacking repeat A, or no *XIST* (vector sequences only) showed no change in allelic ratio upon DOX induction; however, in these cell lines the DNA ratio showed an equivalent allelic DNA ratio, reflecting karyotypic instability of the HT1080 line. There was a significant allelic silencing of *BCL6* with the construct containing only repeat A; however, the reduced silencing seen with this construct suggests that additional sequences are required for the spread of the XIST-induced silencing effect beyond the immediate *XIST* domain.

As repeat A binds the polycomb group 2 proteins that are responsible for trimethylation of H3K27, we asked whether there would be a differential ability of full-length versus repeat A alone to recruit H3K27me3. However, we did not observe any H3K27me3 enrichment by ChIP at the *EGFP*, *Hyg* or the *CLDN16* promoters (Additional file 1: Figure S1). H3K27me3 is a mark of the inactive X, and has been shown to be enriched at the promoters of inactivated genes [23]; however, given that the silencing we have observed in this system is reversible ([13] and data not shown), it is perhaps not surprising that this heritable mark of silent chromatin is not recruited. Similarly, we had previously shown that there was no recruitment of DNA methylation in this reversible system [13]. A similar inducible transgene in mice had identified a developmental window during which inactivation could occur [24], yet we observe induction of silencing in our somatic cell model; possibly reflecting a more epigenetically dynamic state to these cancer-derived cells, or differences in the genes being examined, as we observed variability between genes in their ability to be silenced. By recapitulating XIST-induced gene silencing, but not requiring sequences involved in the spread of XIST, the A repeat construct exposes the most basal aspects of XIST silencing function. To identify the minimal functional unit for silencing, we further dissected the repeat A sequences.

### Repeat A monomers contribute additively to silencing

**Figure 2:**
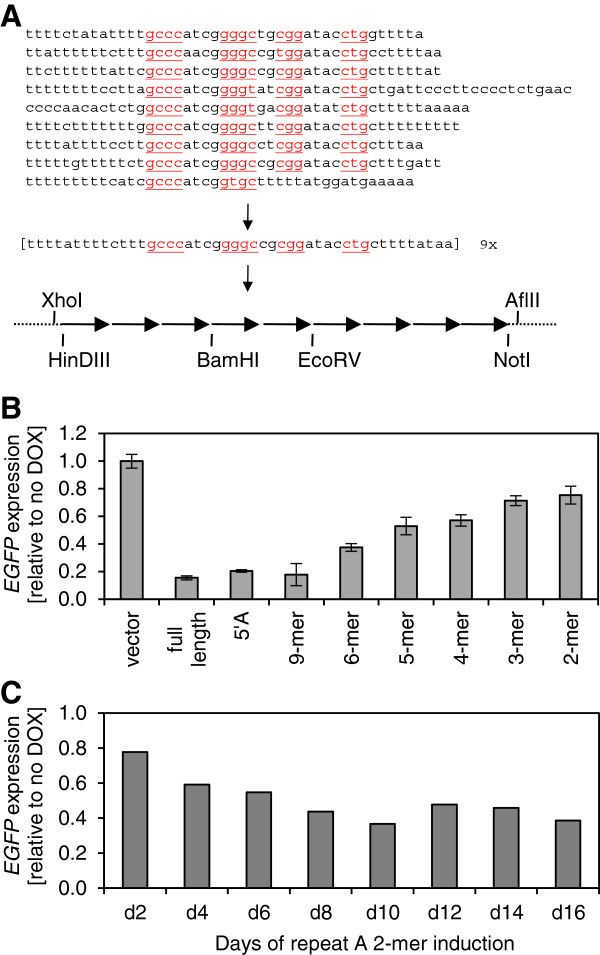
**Repeat A monomers additively contribute to silencing. (A)** Human repeat A sequence consists of 8.5 copies of a well-conserved CG-rich core and T-rich spacer sequences. Palindromic sequences hypothesized to form a secondary structure are underlined. Artificial repeat A was constructed as a 9-mer repetition of consensus monomer sequence and restriction enzyme sites were introduced to allow for the creation of shorter constructs. **(B)** Enhanced Green Fluorescent Protein gene *(EGFP)* expression following five days of transgene induction as measured by qRT-PCR, relative to d0 and normalized to changes in expression caused by induction of the vector alone and to *ACTB* expression for two biological replicates. **(C)***EGFP* expression was measured by flow cytometry every 2 days for 16 days following induction of repeat A 2-mer. Data are normalized to *EGFP* expression in cells that were not induced with DOX.

In order to further characterize the link between repeat A sequence and its silencing ability, we generated an artificial repeat A construct that tested the potential impact of sequence variations in the individual monomers, which are particularly prevalent in the T-rich linker regions. This artificial repeat A consisted of a nine-fold repetition of a 46 bp consensus monomer sequence, and contained restriction enzyme sites in the T-rich stretches to allow for the creation of constructs with reduced numbers of repeats (Figure 2A). Flow cytometry and q-PCR showed that the artificial repeat A silenced *EGFP* to the same extent as full-length *XIST* or human repeat A constructs. Since variability within the individual repeats and spacer regions did not contribute to silencing, we were then able to test the silencing ability of constructs with fewer repeats. Transgenes harboring two to six repeat A monomers were functional, with an approximately linear relationship between the number of repeats and their silencing ability (Figure 2B). Silencing induced by the repeat A 2-mer gradually increased between day 2 and approximately day 8; however, longer induction of the repeat A 2-mer did not promote further *EGFP* down-regulation (Figure 2C).

These observations provide strong evidence that the silencing of an adjacent *EGFP* reporter is achieved through an additive effect of repeat A monomers, with even a 2-mer repeat A inducing partial *EGFP* silencing. The number of repeat A units was previously reported to correlate with the ability of *Xist* to induce silencing in differentiating mouse ES cells [14]. Also, in agreement with a previous report on mouse Xist [14], artificial repeat A retains full silencing potential when compared to human repeat A, suggesting that neither sequence variation within the CG-rich core nor the varying length of the T-rich spacers in individual repeat A monomers is essential for *XIST* function. The remarkable ability of a construct with only two repeats to silence *EGFP* in a reproducible and statistically significant fashion provided us with a well-defined template for further dissection of the relationship between repeat A sequence and its silencing ability.

**Figure 3:**
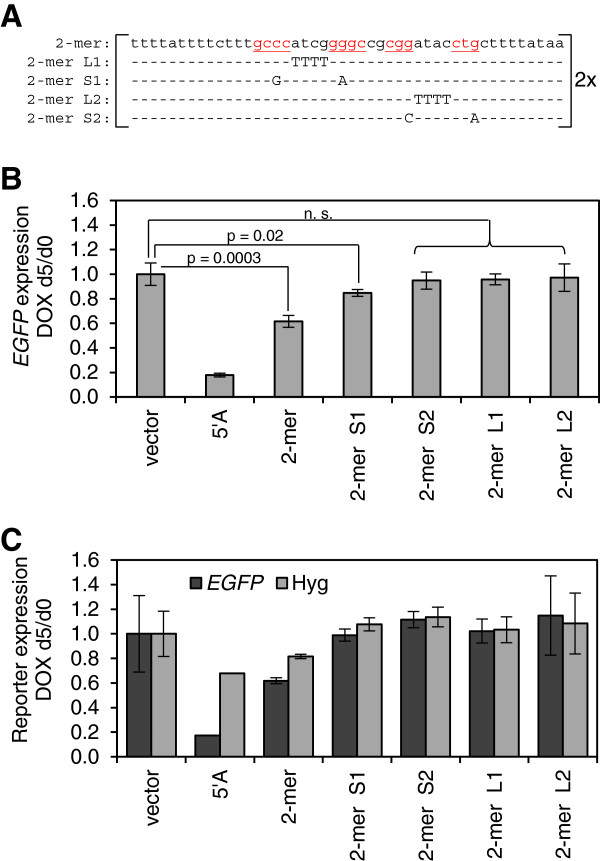
**Mutation of the core repeat A sequences abrogates its silencing ability. (A)** Sequence of the canonical repeat A monomer and four mutant constructs that were created to target the hypothesized repeat A hairpins. Underlined sequences correspond to stem 1 and stem 2. Dashes indicate no sequence change. **(B)** Mean Enhanced Green Fluorescent Protein gene *(EGFP)* expression following five days of transgene induction, measured by flow cytometry, relative to d0 (two-tailed paired *t*-test). Error bars indicate ± 1 S.D. of two single-cell clones. **(C)***EGFP* and hygromycin (*Hyg*) gene expression following five days of transgene induction, measured by qRT-PCR, relative to d0 and normalized to *ACTB* expression for two independent single-cell clones.

The core repeat A sequence consists of two palindromes; the first potentially allowing for perfect C-G pairing linked by ‘ATCG’ and the second involving C-G pairing as well as a G-U pair linked by ‘ATAC’ with the T-rich stretches serving as spacers [14]. While alternative structures have since been proposed, for simplicity, we refer to the four components of CG-rich consensus core as stem 1 (S1), loop 1 (L1), stem 2 (S2) and loop 2 (L2). We initially created a variant of the 2-mer repeat A in each of these elements in order to probe their role in *cis*-silencing of *EGFP* (Figure 3A). Mutations of L1, S2 and L2 completely ablated the transgenes’ ability to silence *EGFP*, as measured by flow cytometry of two representative clones for each mutation, compared to a canonical repeat A 2-mer (Figure 3B). Analysis by qRT-PCR showed the same trends and allowed examination of the *Hyg* gene (Figure 3C); however, flow cytometry affords considerably greater sensitivity as 30,000 events were combined into each datapoint. Mutation of S1 resulted in partial abolition of *EGFP* silencing. Thus, the most conserved regions of *XIST* both among the individual repeats in human (Figure 2A) and among different species (Additional file 2: Figure S2), the CG-rich palindromes and their intervening ‘ATCG’ and ‘ATAC’ sequences, are critical for *XIST* function. All of the previously proposed structures predict the existence of an ‘ATCG’ loop and mutation to ‘TTTT’ in our system completely abolished human repeat A function. Similarly, mutation to ‘TAGC’ in mice has been shown to partially abolish *Xist* function [14], suggesting that the sequence of the tetraloop, and not just its presence, is critical for *XIST/Xist* function.

**Figure 4:**
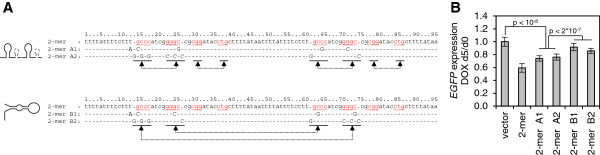
**Stem-loop 1 structure is required to maintain repeat A silencing ability.** Silencing ability of 2-mer repeat A construct is retained when forced to form stem-loop 1 structure, but abrogated when pairing between the monomers is enforced. **(A)** Sequence of the canonical repeat A 2-mer and four mutant constructs that either enforce formation of stem-loop 1 (A1, A2) or an alternative folding (B1, B2) of repeat A sequences, as indicated by schematics. Dashes indicate no change in sequence. **(B)** Mean Enhanced Green Fluorescent Protein gene *(EGFP)* expression following five days of transgene induction, measured by flow cytometry, relative to d0 (two-tailed paired *t*-test). Error bars indicate ± 1 S.D. of two independent single-cell clones and a total of seven biological replicates.

The palindromic nature of the repeat A core sequences strongly suggests their involvement in forming a distinct secondary RNA structure. Several alternative but mutually exclusive structures were previously proposed in which the CG-rich palindrome encompassing the ‘ATCG’ tetraloop (‘stem 1’) may either form a hairpin with pairing within each repeat A monomer [14,21,22] or pairing between two separate monomers [16]. The ability of the repeat A 2-mer to reproducibly induce gene silencing allowed us to use mfold, an RNA structure prediction algorithm [25], to design repeat A mutants that would compare the silencing effectiveness when inter- or intra-repeat pairing was enforced. We found that modeling mutated constructs of larger than 2-mer repeat A structures was highly unreliable as multiple structures of similar minimum free energies (dG) were predicted. We designed a quartet of mutations in the 2-mer repeat A that were predicted to enforce pairing either within (A1, A2) or between (B1, B2) each monomer (Figure 4A and Additional file 3: Figure S3). The repeat A 2-mer mutations were constructed so that a single prominent structure was predicted to fold with either higher (A1, B1), or lower (A2, B2) dG, compared to the unmodified repeat A 2-mer.

Measured by flow cytometry, the mutants predicted to enforce pairing within each monomer functioned better than those enforcing the interaction between the monomers; although none of the four mutants silenced *EGFP* as efficiently as the canonical repeat A 2-mer (Figure 4B), suggesting that there may be more complex structures involved. While the differences in *EGFP* expression were relatively subtle due to a limited silencing effect of the repeat A 2-mer, they were highly statistically significant and equivalent results were obtained for two single-cell clones of independent integrations and a total of seven biological replicates. More representative structures containing greater than two repeat units were not tested as they could not be predicted to reliably form only a single thermodynamically favored structure. However, given the number of eutherian genome sequences that have now been assembled, we turned instead to a characterization of the full repeat A sequences that are available in genome databases.

### Survey of repeat A mutations shows strong preference for stem 1 and mild preference for stem 2 formation

Taking advantage of the increasing number of sequenced mammalian genomes, we generated an alignment of repeat A sequences from 27 mammalian species (Additional file 2: Figure S2A). Repeat A consists of 24 bp-long CG-rich core sequences separated by approximately 20 to 50 bp-long T-rich spacers. The CG-rich core is formed by two palindromes, each of which is broken by four bp-long sequences. As expected, repeat A was well conserved, in particular within the CG-rich core sequences (Additional file 2: Figure S2B). Interestingly, 22/27 mammalian *XIST* sequences contained either eight or nine monomers of repeat A, and at least one of the remaining five was incomplete across the region, supporting the need for eight monomers to achieve full XIST functionality.

**Figure 5:**
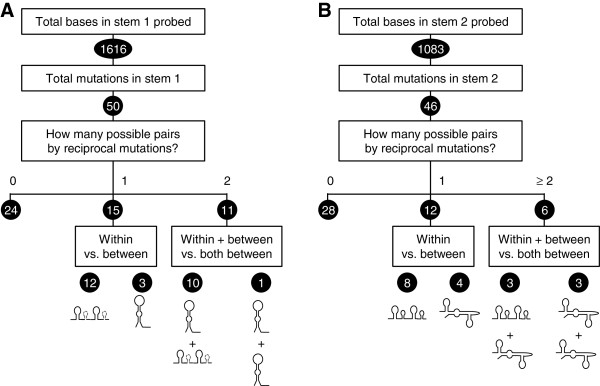
**Compensatory changes in putative stems of repeat A hairpin sequences of 27 mammals. (A)** All *bona fide* repeat A core sequences that deviated in sequence from the canonical stem 1 sequence were categorized by their potential to form a base pair with a reciprocally-mutated base within the same repeat A unit or within another unit. **(B)** As in (A), but stem 2 is analyzed.

Of the defined stem-loop structures, loop 1 showed the highest frequency of deviation from the canonical ‘ATCG’ sequence, with approximately 10% (20/202) of repeat A units harboring an ‘AACG’ tetraloop instead (Additional file 2: Figure S2). To ask whether there was an evolutionary preference for reciprocal mutations that supported the formation of an intra- or interloop configuration we examined deviations from the canonical stem sequences across the *bona fide* monomers of the 27 mammals (Figure 5 and Additional file 4: Figure S4). Despite the strong conservation there were 50 stem 1 changes, allowing us to determine whether fully complementary double-stranded sequences could form due to existing reciprocal mutations either within the same unit, or in another unit of the same species. Of the 50 stem 1 mutations we analyzed, 24 could not be linked with a reciprocal mutation; 12 of the remaining 26 mutations were accompanied by a reciprocal mutation exclusively within the same unit; and a further 10 could pair either within the same unit, or with another unit (Figure 5A). These findings strongly argue in favor of the predicted stem-loop 1 formation. Survey of stem 2 mutations uncovered 46 deviating repeat A units, 28 of which could not pair with any reciprocal mutation (Figure 5B). Of the remaining 18 mutations, 8 could exclusively form a stem-loop by pairing within each unit, with a further 3 allowing for pairing either within a unit or with other units (Figure 5B). While the propensity of the stem 2 region to harbor reciprocal mutations retaining stem-loop 2 formation is less striking than for stem 1, it is still remarkably high, arguing either that stem 2 indeed forms a stem-loop by pairing within each unit, or that repeat A structure involves a combination of both modes of pairing.

Several secondary structures of repeat A have been proposed based on analysis of repeat A mutants [14], NMR data, [21,22] and RNase footprinting and fluorescence resonance energy transfer data [16]. The first palindrome was suggested to form either a hairpin by pairing within each monomer [14,21,22] or, alternatively, to pair between monomers [16]. Both our targeted mutations in an artificial 2-mer repeat A construct, as well as our assessment of evolutionary sequence conservation, support the intra-repeat pairing model, consistent with outcomes observed in mice [14], that the first palindrome indeed forms a stem to expose the ‘ATCG’ tetraloop. The mutations we introduced to the second palindrome also resulted in a complete loss of silencing by *XIST* (Figure 3), supporting the importance of these sequences; however, these mutations did not directly address secondary structure formation. While the second palindrome was proposed to pair within each monomer to form the second stem-loop [14], recent studies suggest that the secondary structure may rather involve pairing between individual repeat A monomers [21,22] or with the T-rich spacers [16]. Our assessment of evolutionary sequence conservation provides evidence in favor of second stem-loop formation, though the frequency of compensatory mutations is less striking than observed for stem-loop 1.

## Conclusions

We utilized a single-copy FRT integration site to generate DOX-inducible *XIST*cDNA integrations allowing the delineation of the repeat A monomers as the minimal functional unit that additively contributes to gene silencing. The ability of only two copies of repeat A to reproducibly silence a flanking *EGFP* reporter gene allowed for further dissection of repeat A sequence to elucidate the relationship between repeat A structure and function. Disruption of either the putative stems or loops of the repeat A abrogated silencing, and mutations of the first palindrome to enforce pairing within a repeat, or between the first and second repeat, supported models that the first palindrome forms a hairpin. An evolutionary analysis of sequence changes within the palindromes allowed assessment of intra- versus inter-repeat pairing in full-length XIST sequences. Again, the model of intra-repeat pairing was favored. The intricate set of events that ultimately lead to X-chromosome inactivation in female mammals remains a vanguard to mammalian epigenetic research. By focusing only on the ability to silence a proximal reporter we have reduced the complexity of deciphering the critical roles of *XIST*. We demonstrate that a mere 94 bp-long sequence of repeat A can silence flanking reporter genes, but not more distal endogenous genes that are silenced by induction of the full-length XIST RNA. Further data on the relationship of repeat A sequence and function will provide a foundation for elucidating the yet unclear connection between the sequence of long non-coding RNAs, like XIST/Xist, and their ability to silence chromatin.

## Methods

### Construct generation

The artificial repeat A construct and its shorter derivatives and mutants were synthesized by GeneArt (now Life Technologies Inc, Burlington, ON, Canada) and cloned into the pcDNA5/FRT/TO plasmid (Life Technologies Inc.) using standard techniques. HT1080 cells were transfected as described previously [13]. Mfold server version 2.3 was used to predict secondary RNA structures (http://mfold.rna.albany.edu) of new constructs.

### Cell culture

Clones harboring single-copy FLP-mediated integrations of XIST constructs into HT1080 fibrosarcoma cell lines were generated and cultured as described previously [13]. The XIST transgenes were induced by doxycycline (1 μg/mL) and cell culture medium was changed every 24 hours.

### Identification of the transgene integration site

Inverse PCR utilizing primers complementary to a sequence within the integrated pEGFP-N1 plasmid (Life Technologies) was used to identify the precise integration site of the *XIST - EGFP* transgene in the HT1080 2-3-0.5 + 3#4 cell line. PCR primer sequences are listed in Additional file 5: Supplementary methods.

### qRT-PCR

RNA was isolated from frozen cell pellets by TRIZOL (Life Technologies Inc.) and treated with DNase I (Roche Diagnostics, Laval, QC Canada) according to the manufacturers’ recommendations. Following phenol-chloroform extraction, RNA concentration was assessed by spectrophotometry and 0.5 to 2.5 μg of RNA was reverse-transcribed by M-MLV reverse transcriptase (Invitrogen). Fermentas HS Taq (Thermo Scientific, Waltham, MA USA) and EvaGreen (Biotium Inc., Hayward CA USA) were used in quantitative PCR under the following conditions: 5 minutes 95°C, 40x (15 sec. 95°C, 30 sec. 60°C, 60 sec. 72°C). PCR primer sequences are listed in Additional file 5: Supplementary methods.

### Flow cytometry

HT1080 cell pellets were washed with PBS and resuspended in 0.5 mL of PBS supplemented with 10% FCS. A total of 30,000 events were recorded using an LSRII flow cytometer (BD Biosciences, Mississauga, ON, Canada). Mean fluorescence intensity of EGFP was assessed by using a combination of 488 nm laser excitation and a 530/30 nm bandpass filter.

### Allelic discrimination by pyrosequencing

A total of 2 μL of cDNA was added to a standard 25-μL pyrosequencing reaction containing 1 × PCR buffer (QIAGEN, Valencia, CA, USA), 0.2 mM dNTPs, 0.625 unit Hot Start *Taq* DNA polymerase (QIAGEN), 0.25 μM forward primer and 0.25 μM reverse primer. PCR conditions were: 95^o^ for 15 minutes, 35 cycles of 94° for 30 sec, 56.3° or 58.3° for 30 sec (see Supplementary table), 72° for 30 sec, and finally 72° for 10 minutes. Template preparation for pyrosequencing was done according to the manufacturer’s protocol, using 10 to 15 μl of PCR products.

### Analysis of repeat A core in mammals

Repeat A sequences in a panel of mammalian species were identified using a combination of BLAST, BLAT and *in silico* PCR searches of mammalian genomes available through NCBI (http://blast.ncbi.nlm.nih.gov) and ENSEMBL (http://www.ensembl.org/Multi/blastview) databases and the UCSC genome browser (http://genome.ucsc.edu). A table listing the accession numbers or genomic locations of repeat A sequences is provided in Additional file 5: Supplementary methods. Sequences were aligned using clustalw2 (http://www.ebi.ac.uk/Tools/msa/clustalw2) and screened to exclude all non-*bona fide* repeat A CG-rich core sequences from further analyses. CG-rich core sequences that contained bases deviating from the canonical sequence of either stem 1 or stem 2 were identified. Finally, we tested whether such a mutation was reciprocated by a mutation within the same repeat A unit, or in all other repeats of that species.

## Abbreviations

ChIP: Chromatin immunoprecipitation; DOX: Doxycycline; EGFP: Enhanced Green Fluorescent Protein gene; ES: Embryonic stem; FCS: Fetal calf serum; Hyg: Hygromycin gene; ncRNA: Non-coding RNA; PBS: Phosphate-buffered saline.

## Competing interests

The authors declare no financial or non-financial competing interests.

## Authors’ contributions

JM designed the study, carried out the cloning and molecular genetic studies, and drafted the manuscript. SELB generated the cell lines described and reviewed the manuscript. CY and AMC contributed to the molecular genetic studies and data analysis, and revised the manuscript. CJB participated in the study design and co-ordination, data interpretation and manuscript preparation. All authors read and approved the final manuscript.

## Supplementary Material

Additional file 1: Figure S1 ChIP for H3K27me3 at silenced promoters. ChIP for H3K27me3 at the *EGFP*, *CLDN16* (2 locations, P1 and P2) and *Hyg* (2 locations, P1 and P2) promoters that are shown to be silenced by DOX-induced expression of *XIST*. H3 shows pull-down for all promoters, while IgG shows limited pull-down. *MYT1,* a silenced gene, is a positive control for H3K27me3 recruitment, and the active *APRT* gene is used as a negative control. Click here for file

Additional file 2: Figure S2 Alignment of repeat A sequences in 27 mammals. **(A)** Sequence alignment of repeat A region in 27 mammalian species. Black circles mark sequences that were not considered bona fide repeat A units and were thus excluded from further analyses. **(B)** Sequence conservation of 202 core repeat A units among 27 mammalian species. Lines on the X axis depict (from top to bottom) the position of bases, percent of units that deviate from canonical sequence, the canonical sequence and arrows corresponding to bases forming the hypothesized stem 1 and stem 2. Click here for file

Additional file 3: Figure S3 *In silico* prediction of repeat A mutant structure. Structures and free energies of 2-mer repeat A and mutants created to enforce pairing within each monomer (A1, A2) or between the two monomers (B1, B2) predicted by mfold. Bases diverging from the canonical repeat A sequence are capitalized and highlighted. Click here for file

Additional file 4: Figure S4 Analysis of repeat A sequences in 27 mammals. **(A)** Illustration of the approach used to analyze repeat A sequence alignment data in Figure 5A, B and Additional file 3: Figure S3B, C. **(B)** Analysis of reciprocal mutations in the stem 1 of individual repeat A units. The table depicts the number of occurrences when mutation in a repeat A unit would allow pairing due to the existence of a reciprocal mutation within the same unit (highlighted), in a different unit or when no reciprocal mutation exists in the species’ repeat A (listed in the last column). (**C**) As in B), but stem 2 is analyzed. Click here for file

Additional file 5 **Supplementary methods.** List of accession numbers or sequence coordinates of repeat A sequences used in sequence analyses and table of primer sequences and ChIP methods. Click here for file
