# Supplementary material for: XIST-induced silencing of flanking genes is achieved by additive action of repeat a monomers in human somatic cells
Source: Epigenetics Chromatin. 2013 Aug 1;6:23. doi: 10.1186/1756-8935-6-23 (PMC3734131; doi:10.1186/1756-8935-6-23)
Supplement: Additional file 1: Figure S1 — ChIP for H3K27me3 at silenced promoters. ChIP for H3K27me3 at the EGFP, CLDN16 (2 locations, P1 and P2) and Hyg (2 locations, P1 and P2) promoters that are shown to be silenced by DOX-induced expression of XIST. H3 shows pull-down for all promoters, while IgG shows limited pull-down. MYT1, a silenced gene, is a positive control for H3K27me3 recruitment, and the active APRT gene is used as a negative control. [file 1756-8935-6-23-S1.pdf]

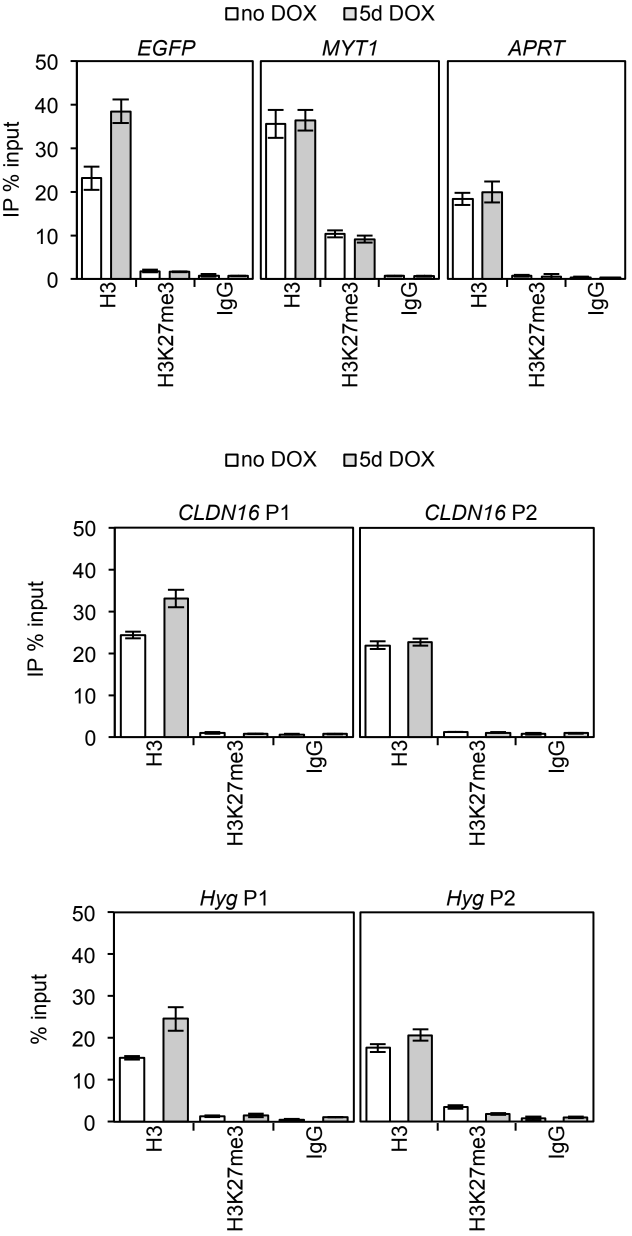

Figure S1. ChIP for H3K27me3 at silenced promoters.
ChIP for H3K27me3 at the EGFP, CLDN16 (2 locations, P1 and P2) and Hyg (2 locations, P1 and P2) promoters which are shown to be silenced by DOX-induced expression of XIST. H3 shows pull-down for all promoters, while IgG shows limited pull-down. *MYT1,* a silenced gene, is a positive control for H3K27me3 recruitment, and the active *APRT* gene is used as a negative control.
