# Supplementary material for: XIST-induced silencing of flanking genes is achieved by additive action of repeat a monomers in human somatic cells
Source: Epigenetics Chromatin. 2013 Aug 1;6:23. doi: 10.1186/1756-8935-6-23 (PMC3734131; doi:10.1186/1756-8935-6-23)
Supplement: Additional file 2: Figure S2 — Alignment of repeat A sequences in 27 mammals. (A) Sequence alignment of repeat A region in 27 mammalian species. Black circles mark sequences that were not considered bona fide repeat A units and were thus excluded from further analyses. (B) Sequence conservation of 202 core repeat A units among 27 mammalian species. Lines on the X axis depict (from top to bottom) the position of bases, percent of units that deviate from canonical sequence, the canonical sequence and arrows corresponding to bases forming the hypothesized stem 1 and stem 2. [file 1756-8935-6-23-S2.pdf]

A

```
                                                       --->.#1.<---  -->.#1.<-
Mus musculus                ----------TGTTTATATAT-TCTT-GCCCATCGGGGCACGGATACCT 38
Rattus norvegicus           -----------TTTTACATTT-TTTTTGCCCATCGGGGCACGGATACCT 38
Ellobius lutescens          -CTTTCTTTTATTCCATCTCTGTTTTTGCCCAT-GGGGCTACAGATGACT 48
Equus caballus              -----------CCTTTTCTGTATTTT-GCCCATCGGGGCTGCGGATACCT 38
Pan troglodytes             -----------TCTTTTCTATATTTT-GCCCATCGGGGCTGCGGATACCT 38
Gorilla gorilla             -----------TCTTTTCTATGTTTT-GCCCATCGGGGCTGCGGATACCT 38
Pongo pygmaeus              -----------TCTCTTTTATATTTT-GCCCATCGGGGCTGCGGATACCT 38
Homo sapiens                -----------TCTTTTCTATATTTT-GCCCATCGGGGCTGCGGATACCT 38
Macaca mulatta              -----------TCTTTTCTATATTTT-GCCCATCGGGGCTGCGGATACCT 38
Callithrix jacchus          -----------TCTTTTCTATATTTT-GCCCATCGGGGCTGCGGATACCT 38
Echinops telfairi           GGCGGCTCTCTTCTTTCTTGTATTTT-GCCCATCGGGGCTGCGGATACCG 51
Cavia porcellus             --------------------------------------------------      ●
Tursiops truncatus          --------------------------------------------------      ●
Oryctolagus cuniculus       -------------------------------------------------A 1    ●
Erinaceus europaeus         -----------TACTTTTTCTATTTT-GCCCATCGGGGCTGTGGATAACA 38
Sorex araneus               CTGTGGATAATTGGTATGATTATTT--GCCCAGCGGGGCTGTGGATGCGT 74
Felis catus                 -----------CCTTTTCTACATTTT-GCCCATCGGGGCTGTGGATACCA 38
Bos taurus                  -----------TTTTCTATATTATTT-GCCCATCGCGGCTGTGGATACCT 38
Sus scrofa                  -----------TTTTCTGTGTATTAT-GCCCATCGGGGCTGTGGATACCT 38
Tupaia belangeri            -----------ATTTATGTATATTTT-GCCCATCGGGGCTGAGGATACCT 38
Microcebus murinus          -----------CTTTTTCTATATTTG-GCCCATCGGGGCTGCGGATATCT 38
Canis lupus familiaris      -----------TTTTTTCTATATTTT-GCCCATCGGGGCTGCGGATACCG 38
Ailuropoda melanoleuca      -----------TATATTCTATATTTT-GCCCATCGGGGCTGCGGATACCG 38
Vicugna pacos               TTTTCCCCCCTCCTTTTCTATATTTT-GCCCATCGGGGTTGTGGATACCT 99
Tarsius syrichta            --------------------------------------------------      ●
Myotis lucifugus            --------------------------------------------------      ●
Pteropus vampyrus           --------------------------------------------------      ●

                            -
Mus musculus                GTGTGTCCTCC----------------------------------CCGCC 54
Rattus norvegicus           GTGTGTCCTCC----------------------------------CAGCC 54
Ellobius lutescens          GCATGTACTCCTT-------------------------CCCCGTCCCTTC 73
Equus caballus              G---ATCTTCT----------------------------------TATTA 51
Pan troglodytes             GGTTT-------------------------------------TATTATTT 51
Gorilla gorilla             GGTTT-------------------------------------TATTATTT 51
Pongo pygmaeus              GGTTT-------------------------------------TATTATTT 51
Homo sapiens                GGTTT-------------------------------------TATTATTT 51
Macaca mulatta              GGTTT-------------------------------------TATTATTT 51
Callithrix jacchus          GGTCT-------------------------------------TA---TTT 48
Echinops telfairi           GATTT-------------------------------------TTTCATTC 64
Cavia porcellus             ------------------------------------------CATTTTAT 8
Tursiops truncatus          ------------------------------------------CCTTTTCT 8
Oryctolagus cuniculus       CATTTCCATATTTTGCCCATCGGGGCTATGGATACCTGGTTTTATTATTT 51
Erinaceus europaeus         GGTTT-----GAT-TATTAACATATT-----GCCCA---ACGGGGCTGTG 74
Sorex araneus               GACTTTTAGAGAT-TTTATATATATTTGGATGTACATGTACATGTATGTA 123
Felis catus                 GGTTTT-----AT-TATCATTTTTTTC----ACCCA---ACGGGGCTGTG 75
Bos taurus                  GGTTTTAGTGTGTATATAGATAGGTAGATAGATATATTATTATTGTTATT 88
Sus scrofa                  GGTTTTAATA--------------------------ATTGTTATT--TATT 61
Tupaia belangeri            GTTTTTTAATTTTTTTTTCACTTTTTG----TTTTTGGTTTCTTTTTTTC 84
Microcebus murinus          AGTTTCA----------------------------------TTATTGTTT 54
Canis lupus familiaris      GGTTTATTATTA----------------------------------TTAT 55
Ailuropoda melanoleuca      GGTTTCACTATTAGT------------------------AGTAGTATCAT 64
Vicugna pacos               GGTTTTAATATTT-------------------------TTTGTTTTGTGT 124
Tarsius syrichta            --------------------------------------------AGTTGA 6
Myotis lucifugus            -------------------TGATTTAGTTCTCTATTTCCTCCACTTTTCT 31
Pteropus vampyrus           ------------------------------------------TTTTTTCT 8

                                   --->.#2.<---   -->.#2.<--
Mus musculus                ATTCCATGCCCAACGGGGT-TTTGGATACTTA-CC--------TGCCTTT 94
Rattus norvegicus           ATTCCATGTCCAGCTGGGC-TTGGGATACTTAACC--------TGCCTTT 95
Ellobius lutescens          ACTCCGTGCCCAGTGGGGC-TGTGGATACTTACCCGCTTTTAATTCGTTT 122
Equus caballus              TTTTTTTGCCCAACGGGGC-TGTGGATACCTG-------------CCTTT 87
Pan troglodytes             TTTCTTTGCCCAACGGGGC-CGTGGATACCTG-------------CCTTT 87
Gorilla gorilla             TTTCTTTGCCCAACGGGGC-CGTGGATACCTG-------------CCTTT 87
Pongo pygmaeus              TTTCTTTGCCCAACGGGGC-CGTGGATACCTG-------------CCTTT 87
Homo sapiens                TTTCTTTGCCCAACGGGGC-CGTGGATACCTG-------------CCTTT 87
Macaca mulatta              TTTCTTTGCCCAACGGGGC-TGTGGATACCTA-------------CCTTT 87
Callithrix jacchus          TTTCTTTGCCCAACGGGGC-CGTGGATACCTG-------------CCTTT 84
Echinops telfairi           TTCCTTAGCCCATCGGGGT-TGTGGATAGCTG-------------CCTTA 100
Cavia porcellus             ATATTTTGCCCATCGGGGC-CGTGGATACCTG-------------CTTTA 44
Tursiops truncatus          ATATTTTGCCCATCGGGGC-TGTGGATACCTG-------------GTTTT 44
Oryctolagus cuniculus       TTTCTTTGCCCAACAGGGT-TCTAGCTACCTG-------------TCTTA 87
Erinaceus europaeus         GATACCTGC-CTTATAGAT-------CACGAC-------------TATCT 103  ●
Sorex araneus               CATATGTATGCATATACATACGTATGTATGTG-------------TATAT 160
Felis catus                 GATACCTGCGTTTTAATTC-------TTTTCT-------------TTTAT 105  ●
Bos taurus                  ATTTTTTACCCAACGGGGT-CATGGATACCTG-------------CCTTT 124
Sus scrofa                  --TTTTTGCCCAACGGGGC-CGTGGATACCTG-------------CCTTT 95
Tupaia belangeri            CCCCTTTGCCCAACGGGGC-CGTGGATACCTG-------------CCTCT 120
Microcebus murinus          TTTCTTTGCCCAACGGGGC-TGTGGATACCTG-------------CCTTT 90
Canis lupus familiaris      TATTATTGCCCAACGGGGC-TGTGGATACCTG-------------CCTTT 91
Ailuropoda melanoleuca      TATTTTTGCCCAACGGGGC-TGTGGATACCTG-------------CCTTT 100
Vicugna pacos               ATTTTTTGCCCAACGGGGC-CATGGATACCTG-------------CTTTT 160
Tarsius syrichta            CATTTCTGCTTCTTGTAGT--TTAGTTTTCTA-------------TTCCA 41   ●
Myotis lucifugus            ATATTTTGCCCATCAGGGT-TGCGGATACCTG--------------ATTT 66
Pteropus vampyrus           ATATTTTGCCCATCGGGGC-CGCGGATACCTG--------------CTTT 43
```

```
                                                             --->.#3.
Mus musculus                TCATTCTTTTTTTTTCTTATTATTTTTTTT---TCTAAACTTGCCCATCT 141
Rattus norvegicus           TAATCCTTTTTTCTTCTTCTTACTTTTCTTCT-TCTAAACTTGCCCATCT 144
Ellobius lutescens          TGGTTTTTCCCTCCTTCTCCTCTTCTTTTTCT-TCTAAATTTGCCCATCT 171
Equus caballus              TAATTTTTTTTTTTTTT--------------------AATTTGCCCATCG 117
Pan troglodytes             TAATTCTTTTTT-ATTC-------------------------GCCCATCG 111
Gorilla gorilla             TAATTCTTTTTT-ATTT-------------------------GCCCATCG 111
Pongo pygmaeus              TAATTCTTTTTT-ATTC-------------------------GCCCATCG 111
Homo sapiens                TAATTCTTTTTT-ATTC-------------------------GCCCATCG 111
Macaca mulatta              TAATTCCTTTTT-ATTC-------------------------GCCCATCG 111
Callithrix jacchus          TAATTCTTTTTTTATTT-------------------------GCCCATCG 109
Echinops telfairi           TTATATTTTTCCTTTTCA----------------------TCGCCCATCG 128
Cavia porcellus             AAT-------------TTA--------------------TTTGCCCATCG 61
Tursiops truncatus          AATATTGTTAT-----TTT--------------------TTTGCCCATCG 69
Oryctolagus cuniculus       ATTCCTTTTTTAAAGAAAA--------------------TTAGCCCACCG 117
Erinaceus europaeus         G-ATCATTATTCTTTT-----------------------TTTGTCCATCG 129
Sorex araneus               GCATTAATGTGTATCT-----------------------TTTGCCCATCG 187
Felis catus                 ---TTATTTTTTTTAA-----------------------TTTGCCCATCG 129
Bos taurus                  TATTTATTTTTTTTTTAA---------------------TTTGCCCATCG 153
Sus scrofa                  TAATTCTTTTTTTAAAGAC--------------------TTTGCCCATCG 125
Tupaia belangeri            TAACTTTTCTCTTTTTAA---------------------TTTGCCCATCG 149
Microcebus murinus          TAATTCTTTTTCTTTTTTAAA-----------------ATTTGCCCATCG 123
Canis lupus familiaris      TAATTCTTTTTTTTTGTTTGTTTGTTTTT-------AAATTTGCCCATCG 134
Ailuropoda melanoleuca      TAATTCTTTTCTTTTCTTTTCTTTCTTTCTTTTTTAAAATTTGCCCATCG 150
Vicugna pacos               TAATTCTTTTTTTTTTTTAA--------------------TTGCCCATCG 190
Tarsius syrichta            TCACCCCTTTTTCTATAT---------------------TTTGCCCATCG 70
Myotis lucifugus            TATTTATTTACTTTAAAAAA-------------------TGTGCCCATCG 97
Pteropus vampyrus           TAATTTTTTTCCCCCTTAA-----------------------GCCCATCG 70

                            <---  -->.#3.<--
Mus musculus                GGGCTGTGGATACCTGCT---TTTATTCTTTTTTTCTTCT---CCT---- 181
Rattus norvegicus           GGGTTGTGGATACCTGCT---TTTATTCTTTTTTTCTTCTTCTCCT---- 187
Ellobius lutescens          GGGCTGTGGATACCTGCT---CTTCTTTTTTT-----------CCT---- 203
Equus caballus              GGGCCACGGATACCTGCG---TTTATTTTTTTTTTCCC-----CCT---- 155
Pan troglodytes             GGGCCGCGGATACCTGCT---TTTTATTT--TTTTTTT-----CCT---- 147
Gorilla gorilla             GGGCCGCGGATACCTGCT---TTTTATTT--TTTTTT------CCT---- 146
Pongo pygmaeus              GGGCCGCGGATACCTGCT---TTTTATTT--TTTTTTT----TCCT---- 148
Homo sapiens                GGGCCGCGGATACCTGCT---TTTTATTT--TTTTTT------CCT---- 146
Macaca mulatta              GGGCCGCGGATACCTGCT---TTTTATTT--TTTTTTT----TCCT---- 148
Callithrix jacchus          GGGCCGCGGATACCTGCT---TTTTATTA--TTTTTTT----TCCT---- 146
Echinops telfairi           GGGCTGCGGATACCTGCT---TTTAATTTCATTTTTTT----CCCT---- 167
Cavia porcellus             GGGCTGCGGATACCTGCC---TTTTTTCTTTTTTTC------CTCT---- 98
Tursiops truncatus          GGGCCGCGGATACCTGCT---TTTAATTTTTTTTTC------C-CT---- 105
Oryctolagus cuniculus       GGGCTGCGGATACCTGCT---TTTAATTTTTTTTTC------C--T---- 152
Erinaceus europaeus         GGGCCTCGGATACCTGTT---TTTATTTTTTCT-CC------CCCT---- 165
Sorex araneus               GGGCCATGGATACGTGCG---CTAAAAACTTTTATC------CCCT---- 224
Felis catus                 GGGCAGCGGATACCTGCT---TTTAATTTTTTTTTTTTTTCACCCT---- 172
Bos taurus                  GGGCCACGGATACCTGCT---TTTAATTTTTTTTTT----CCCCCT---- 192
Sus scrofa                  GGGCCGCGGATACCTGCT---TTTAATTTTTTTTT------CCCCT---- 162
Tupaia belangeri            GGGCCACGGATACCTGCT---TTTTTATTATTATTA----TTTTCT---- 188
Microcebus murinus          GGGCAGTGGATACCTGCT---TTTTATTTTTTTTTTTT--CCCCCT---- 164
Canis lupus familiaris      GGGCCACGGATACCTGCT---TTTATTTTTTTTT-C------CCCT---- 170
Ailuropoda melanoleuca      GGGCCGCGGATACCTGCT---TTAATTTTTTTTTTC------CCCT---- 187
Vicugna pacos               GGGCCGCGGATACCTGCT---TTTAATTTTTTTTCC------TCCT---- 227
Tarsius syrichta            GGGCTGCGGATACCTGGT---TTTATTATTATTTTTG------TCA---- 107
Myotis lucifugus            GGGCCGCGGATACCTGCT---TTTAATTTTTTTCCCC-----CCCT---- 135
Pteropus vampyrus           GGGCGCGGATACCTGCTGTGTCCCCTCTTCATCCCCAA-TCCCCTAACT 119

                                 --->.#4.<---  -->.#4.<--
Mus musculus                ---TAGCCCATCGGGGCCATGGATACCTGCTTTTTGTAAA-AAAAAAAAA 227
Rattus norvegicus           ---TAGCCCATCGGGGCCATGGATACCTGCTTTTTACCAA-AAAACGCCG 233
Ellobius lutescens          ---TAGCCCATCGGGGCCATGGATACCTGCCTTTTTTTAACAAGAAAACG 250
Equus caballus              ---CAGCCCATCGGGGCCTCGGATACCTGCTTTTTTCCTT-----AAATT 197
Pan troglodytes             ---TAGCCCATCGGGGTATCGGATACCTGCTGATTCCCTTCCCCTCTGAA 194
Gorilla gorilla             ---TAGCCCATCGGGGTATCGGATACCTGCTGATTCCCTTCCCCTCTGAA 193
Pongo pygmaeus              ---TAGCCCATCGGGGTATCGGATACCTGCTGATTCCCTTCCCCTCTGAA 195
Homo sapiens                ---TAGCCCATCGGGGTATCGGATACCTGCTGATTCCCTTCCCCTCTGAA 193
Macaca mulatta              ---TAGCCCATCGGGGCATCGGATACCTGCTGATTCCCTTCCCCTCTGAC 195
Callithrix jacchus          ---TAGCCCATCGGGGCATCGGATACCTGCTGATTCCCTTCCCCTCTGAC 193
Echinops telfairi           ---TAGCCCATCGGGGCCATGGATGCGTGC-GATCTCCTCTCCACCCGA- 212
Cavia porcellus             ---TAGGCCATCGGGGCCT-GGATACCTGCTG-GTACACTAACCCTCC-- 141
Tursiops truncatus          ---TAGCCCATCGGGGCCTCGGATACCTGCTGTGTACCCCCTCTCTCCCC 152
Oryctolagus cuniculus       ---TAGCCCATCGGGGCCTCGGATACCTGCAGTGCCCCCTTTTGCCCCCC 199
Erinaceus europaeus         ---TAGCCCATCGGGGCCTTGGATAGCTGCTGTCCACTTCCTCCCCTCAA 212
Sorex araneus               ---TCGCCCATCCGGGCCTCGGATACCTGCAGTCCGCTCCCTAACCTTAT 271
Felis catus                 ---TAGCCCATCGGGGCCTCGGATACCTGCTGT--GTCTCCTTCCCTC-- 215
Bos taurus                  ---TAGCCCATCGGGGCCTCGGATACCTGCTGTGTACCCCCTCTCTCC-- 237
Sus scrofa                  ---TAGCCCATCGGGGCCTCGGATACCTGCTGTGT-CCCCCTCTTTCT-- 206
Tupaia belangeri            ---TAGCCCATCGGGGCCTTGGATACCTGCTGTGT-CTCCCCCTCCCT-- 232
Microcebus murinus          ---TAGCCCAT-GGGGCAGTGGATGTGT-CCACGCGCCCCCCCCCCCAAC 209
Canis lupus familiaris      ---TAGCCCATCGGGGCCTCGGATACCTGCTGTGCCCCCCCCC------- 210
Ailuropoda melanoleuca      ---TAGCCCATCGGGGCCTCGGATACCAGCTGTGTCCCCCCTCTCTCT-- 232
Vicugna pacos               ---TAGCCCATCGGGGCCTCGGATACCTGCTGTGGCCCCCTTTTCTCCCC 274
Tarsius syrichta            ---TTGCCCAACGGGGCTGTGGATACCTGCCTTATAATTATTATTATTTT 154
Myotis lucifugus            ---TAGCCCATCGGGGCCTCGGATAGCTGCTGTGTCCTCCTTTTCCCC-- 180
Pteropus vampyrus           CTGTAGCCCATCGGGGCCACGGATACCTGCTATTTTTTTTTTTTTC--T-- 165
```

```
                                                    --->.#5.<---  -->.#5.<--
Mus musculus                  AAAAAAAAAAAAACCTTTCTCGGTCCATCGGGACCTCGGATACCTGCGTT 277
Rattus norvegicus             TA-------------TTTCTCGGTCCATCGGGACCTCGGATACCTGCGTT 270
Ellobius lutescens            TA-------------TTTCTTGGTCCATCGGGACCTCGGATACCTGCGTT 287
Equus caballus                TA-------------TTTTTTGGCCCATCGGGGCCTCGGATACCTGCTTC 234
Pan troglodytes               C--------CCCCAACACTCTGGCCCATCGGGGTGACGGATACCTGCTTT 236
Gorilla gorilla               C--------CCCCAACACTCTGGCCCATCGGGGTGACGGATACCTGCTTT 235
Pongo pygmaeus                C--------CCCCAACACTCTGGCCCATCGGGGTGACGGATACCTGCTTT 237
Homo sapiens                  C--------CCCCAACACTCTGGCCCATCGGGGTGACGGATATCTGCTTT 235
Macaca mulatta                C--------CCCCAACACTCTGGCCCATCGGGGTGGCGGATACCTGCTTT 237
Callithrix jacchus            C--------CCCCAACACTCTGGCCCATCGGGGTGACGGATATCTGCTTT 235
Echinops telfairi             C--------CCATGGCCCACGAAGCAAC--AAATAGCAGTACTTTATTAC 252 ●
Cavia porcellus               ------------ATTTT--CTGGGCCATCGGGGCAATGGATACCTGCTTT 177
Tursiops truncatus            A-----------ACCCT--CTGGCCCAACGGGGCAATGGATACCTGCCTT 189
Oryctolagus cuniculus         ------------AAATT--CTGGCCCATCGGGGCAACGGATACCTGTTTT 235
Erinaceus europaeus           A---AATTAACAAATTT--TTGACCCACCGGGGTAACGGATACCTGTTTT 257
Sorex araneus                 AC--AAACATTTAAATGAGCCGGCCCATCAGGGCAACGGATACCTGCTTT 319
Felis catus                   -------CCCTTAACCCT-CTGGCCCATCGGGGCAATGGATACCAGCTTT 257
Bos taurus                    ----------CTAACC----TGGCCCATCGGGGCAATGGATACCTGCCTC 273
Sus scrofa                    ----------CCAACCCC-TTGGCCCATCAGGGTAATGGATACCTGCTTT 245
Tupaia belangeri              ------------AAACTCTCTGGCCCATCGGGGCATAGGATACCAGCTTT 270
Microcebus murinus            T-------------CCTCTCTGGCTGATCGGGGCAACGGATACCTGGTTT 246
Canis lupus familiaris        ----------CCCAACTCCCTGGCCCATCGGGGCAATGGATACCTGCTTA 250
Ailuropoda melanoleuca        ---------CCCCAACCCCCTGGCCCATCGGGGCAATGGATACCTGCTTA 273
Vicugna pacos                 A-------------ACCTCCTGGCCCATCGGGGCAACGGATACCTGCTTT 311
Tarsius syrichta              C---------TTTTTGAAATTCACCCATCGGGGTCACGGATACCTGCTTT 195
Myotis lucifugus              -------------AAACCACTGGCCCACCGGGGCCTAGGATACCTGCTTT 217
Pteropus vampyrus             -------------ATTTTTCTTGCCCATCGGGGCCTCGGATACCTGCTTT 202

                                                                             ---
Mus musculus                  T------AGTCTTT--------------TTTTCCCAT----------GCC 297
Rattus norvegicus             T------AGT--TT--------------TTTTCCCAT----------GCC 288
Ellobius lutescens            T------AGT-------------------CTTTCCTT----------GCC 302
Equus caballus                T------ATTTTTT--------------TTTTTCCTT----------GCC 254
Pan troglodytes               T-----TAAAAATT-------------TTCTTTTTTT---------GGCC 259
Gorilla gorilla               T-----TAAAAATT-------------TTCTTTTTTT---------GGCC 258
Pongo pygmaeus                T-----TAAAAATT-------------TTCTTTTTT----------GGCC 259
Homo sapiens                  T-----TAAAAATT-------------TTCTTTTTTT---------GGCC 258
Macaca mulatta                T-----TAAAAATT-------------TTGTTTTTTT---------GGCC 260
Callithrix jacchus            T-----TAAAAATT-------------TTCTTTTTTT---------GGCC 258
Echinops telfairi             C-----CATCATAT-------------ATATTTTTTT---------GGCC 275
Cavia porcellus               TTTTTTTTTTTTTTTTTAATTATGTGTGTGTTTGTTTGTTTGTTTTGGCC 227
Tursiops truncatus            TTTTTTTTTTTTT--------------AAATTTATTT------TTTGGCC 219
Oryctolagus cuniculus         TGTGGTTTTTTTTGTTTTGTTTT----GTTTTTGAAT-TGGCTTTTGGCC 280
Erinaceus europaeus           TTTTTTTAAAATGT--------------TTTTTTTTTTT-------GGTC 286
Sorex araneus                 TCTTTTCATATAAT--------------TGTTTTCTT---------GGTC 346
Felis catus                   A------AAAAAAG--------------TTCCTTTTT---------GGCC 278
Bos taurus                    TTTTTTAAATGTGT--------------TGTTTTTTTTTTTTTCCTTGCC 309
Sus scrofa                    TTTATTTAAAAAAA--------------AATTTTTTTTG--------GCC 273
Tupaia belangeri              T-----AAAAATAC--------------AATAAAATAAAAAGATTTTGCC 301
Microcebus murinus            T-----TAAAAATC--------------TTTGTTTTT---------GGCC 268
Canis lupus familiaris        A------AAAAAA----------------TTACTTTT---------GGCC 269
Ailuropoda melanoleuca        A------AAAAAAA--------------TTTACTTTTT--------GGCC 295
Vicugna pacos                 TTTTAAAAAAAAAA----------------TTTTTTT----------GCC 335
Tarsius syrichta              T------ATTTTTA--------------TTTTTAATT---------AGCC 216
Myotis lucifugus              T------TTTTTTT--------------TTTCTTCTTTT----TATAGCC 243
Pteropus vampyrus             C------ACTATTT--------------TTTTTCTT-----------GCC 221

                              >.#6.<---  -->.#6.<--
Mus musculus                  CAACGGGGCCTCGGATACCTGCTGTTATTATTTTTT-----TTTCTTTTT 342
Rattus norvegicus             CAACGGGGCCTCGGATACCTGCT-TTA--ATTTTTT-----TTTCTTTTC 330
Ellobius lutescens            CATCGGGGCCTTGGATACCTGCT-TCA-----TTTT-----TTTTCCCTC 341
Equus caballus                CATCGGGGCCTCGGATACCTGCTCTCA--------------TTTTTTTTT 290
Pan troglodytes               CATCGGGGCTTCGGATACCTGCTTTTTTTTTTTTTT---------ATTTT 300
Gorilla gorilla               CATCGGGGCTTCGGATACCTGCTTTTTTTTTTTTTT---------ATTTT 299
Pongo pygmaeus                CATCGGGGCTTCGGATACCTGCTTTTTTTTTTTTTTT--------ATTTT 301
Homo sapiens                  CATCGGGGCTTCGGATACCTGCTTTTTTTTTTTTTA-----------TTT 297
Macaca mulatta                CATCGGGGCTTCGGATACCTGCTTTTATTTTTTATT------------TT 298
Callithrix jacchus            CATCGGGGCTTCGGATACCTGCTTTTATTTTTTATT------------TT 296
Echinops telfairi             CACCCGGGCTGTGGATACCTGATTTTATTTTGTTTTTTTAAAAAAATGTT 325
Cavia porcellus               CACCGGGGCCCTGGATACCTGCTT-----TA----------TTATTTTTT 262
Tursiops truncatus            CATCGGGGCCTTGGATACCTGCTT-----TGATTTT-----TCTTTTTTT 259
Oryctolagus cuniculus         CATCGGGGCCTCGGATACCTGCTTA----TATTTTT-----TTTTTAAAT 321
Erinaceus europaeus           CATCGGGACCCTGGATATATGATTT-----TATT--------AAAACTTT 323
Sorex araneus                 CATCGGGACCCTGGATACCTGCTCG-----GTTT--------TTTTTTTT 383
Felis catus                   CATCGGGGCCTCGGATACCTGCTTT-----TATTA-------TTTTTTTT 316
Bos taurus                    CATCGGGGCCTCGGATACCTGCTTT-----AATT--------TTTTTTTT 346
Sus scrofa                    CATCGGGGCCTCGGATACCTGCTTT-----AATT--------TTTTTTTT 310
Tupaia belangeri              CATCGGGGCCTCGGATACCTGCTTTTCAAAAATTAAAAAAAAAATTTTTT 351
Microcebus murinus            CATCGGGGCTTCGGATACCTGCTTT-----TATTGT--------TTTTTT 305
Canis lupus familiaris        CATCGGGGCTTCGGATACCTGCTTT----TATTTT--------TTTTTTC 307
Ailuropoda melanoleuca        CATCGGGGCCTCGGATACCTGCTTT----TTTTTT--------TTTTTTC 333
Vicugna pacos                 CATCGGGGCCTCGGATACCTGCTTT-----AATTTT--------TTTTTC 372
Tarsius syrichta              CATCGGGGCATCGGATACCTGCTGTGTCCCCCCCAC---------CAACC 257
Myotis lucifugus              CATCGGGGCTTCGGATACCTGCTTTAA------------------TTTTT 275
Pteropus vampyrus             CATCGGGGCCTCGGATACCTGCTTTAAA-------------TTTTTTTTT 258
```

```
                                       --->.#7.<---  -->.#7.<--
Mus musculus                 CTTTTGCCCATCGGGGCTGTGGATACCTGCTTTAAATTTTTTTTTTC--- 389
Rattus norvegicus            CTTTTGCCCATCGGGGCTGTGGATACCTGCTTTAA-TTTTTTTTTTC--- 376
Ellobius lutescens           TTTTTGCCCATCGGGGCTGTGGAAACCTGCTTCA---TTTTTTTTTC--- 385
Equus caballus               TCCTTGCCCATCGGGGCCACGGATACCTGCTTAGATTTTTTTTTTTC--- 337
Pan troglodytes              TCCTTGCCCATCGGGGCCTCGGATACCTGCTTTAATTTTT------GTTT 344
Gorilla gorilla              TCCTTGCCCATCGGGGCCTCGGATACCTGCTTTAATTTTT------GTTT 343
Pongo pygmaeus               TCCTTGCCCATCGGGGCCTCGGATACCTGCTTTAATTTT-------GTTT 344
Homo sapiens                 TCCTTGCCCATCGGGGCCTCGGATACCTGCTTTAATTTTT------GTTT 341
Macaca mulatta               TCCTTGCCCATCGGGGCCTCGGATACCTGCTTTAATTTTT------GTTT 342
Callithrix jacchus           TCCTTGCTCATCGGGGCCTCGGATACCTGCTTTAATTTT---------TT 337
Echinops telfairi            CCCTTGCCCATCGGGGCCTCGGATACCGGCTCTGATTTTTTTCCCCGTTC 375
Cavia porcellus              TCATTGCCCATCGGGGCCTCGGATACCTGCCCTAATTTTTTTTTT----- 307
Tursiops truncatus           CCTTTGCCCATCGGGGCCTCGGATACCTGCTTTAATTTTTTTTTTCC--- 306
Oryctolagus cuniculus        TTTTTGCCCATCAGGGCCTCGGATACCTGCTCTGATTTTTTTTTTTT--- 368
Erinaceus europaeus          CAACTGCCCATCGGGGCAT-GGATACCTGCTTAATTTTGT-TTTTC--- 368
Sorex araneus                TCCTTGCCCATCGGGGCCTCGGATACCTGCTCGCTTTTTTT-TTTTTTTC 432
Felis catus                  CCCTTGCCCATCGGGGCCTTGGATACCTGCTTTATTTATTTATTTTTTTT 366
Bos taurus                   --CTTGCCCATCGGGGCCTCGGATACCTGCTTTAAT---TTTTTTTC--- 388
Sus scrofa                   TCCTTGCCCATCGGGGCCTCGGATACCTGCTTTATTATTTTTTTTTC--- 357
Tupaia belangeri             TTTTGGCCCATCGGGGCTTCGGATACCTGCTTTAATTTTTTTTTGTTGTT 401
Microcebus murinus           CCCTTGCCCATCGGGGCCTTGGATACCTGCTTTTATTATTTTTTTTT--- 352
Canis lupus familiaris       CC-TTGCCCATCGGGGCCTCGGATACCTGCTTTTAATTTTTTTT------ 350
Ailuropoda melanoleuca       CCCTTGCCCATCGGGGCCTCGGATACCTGCTTTAATTTTTTTTTT----- 378
Vicugna pacos                CTCTTGCCCATCGGGGCCTCGGATACCTGCTTTAATTTTTTTTTT----- 417
Tarsius syrichta             CTTTGGCCCATCGGGGCAACGGATACCNNNNNNNNNNNNNNNNNNNNNNN 307
Myotis lucifugus             TTCTTGCCCATCGGGGCCGCGGATACCTGCTTAGATTTTTTTTTTTT--- 322
Pteropus vampyrus            TCCTTGCCCATCGGGGCCGCGGATACCTGCTTAGATTTTTTTTTTA---- 304

                                --->.#8.               <---  -->.#8.<--
Mus musculus                 --ACGGCCCAACG----------------GGGCGCTTGGTGGATGGAAAT 421
Rattus norvegicus            --ACGGCCCATCG----------------GGGCATTTGGTGGATGGAAAT 408
Ellobius lutescens           --CTTGTCCATCG----------------GGGCATTTGGTGGATGGATAT 417
Equus caballus               --ATTGCCCATCG----------------GGGGTTTTTATGGATAGAAAA 369
Pan troglodytes              TTCTGGCCCATCG----------------GGGCCGCGGATACCTGCTTTG 378
Gorilla gorilla              TTCTGGCCCATCG----------------GGGCCGCGGATACCTGCTTTG 377
Pongo pygmaeus               TTCTGGCCCATCG----------------GGGCCGCGGATACCTGCTTTG 378
Homo sapiens                 TTCTG-CCCATCG----------------GGGCCGCGGATACCTGCTTTG 374
Macaca mulatta               TTCTGGCCCATCG----------------GGGCCGCGGATACCTGCTTTG 376
Callithrix jacchus           TTCTGGCCCATCG----------------GGGCCGCGGATACCTGCTTTG 371
Echinops telfairi            TCTGGGCCCATCG----------------GGGCCGCGGATACCTGCTGTG 409
Cavia porcellus              -TT--GCCCATCG----------------GGGCCTTGGATACCTGCTTTA 338
Tursiops truncatus           -TT--GCCCATCG----------------GGGCCGCGGATACCTGCTTAG 337
Oryctolagus cuniculus        -TCTGGCCCATCG----------------GGGCCCCGGATACCTGCTCTG 401
Erinaceus europaeus          --CTTGCCCATCG----------------GGGCCACGGATACCTGCTTAG 400
Sorex araneus                CACTTGCCCATCG----------------GGGCCATGGAT---------- 456 ●
Felis catus                  CCTTTGCCCATCG----------------GGGCTGTGGATACCTGCTTAG 400
Bos taurus                   --CTTGCCCATCG----------------GGGCCGCGGATACCTGCTTAG 420
Sus scrofa                   --CTTGCCCATCG----------------GGGCCGTGGATACCTGCTTAG 389
Tupaia belangeri             TTTTGGCCCATCG----------------GGGCCACGGATACCTGCTCTC 435
Microcebus murinus           TCCTTGCCCATCG----------------GGGCCTCGGATACCTGCTCTC 386
Canis lupus familiaris       -CCTTGCCCATCG----------------GGGCTGTGGATACCTGTTTAG 383
Ailuropoda melanoleuca       -CCTTGCCCATCG----------------GGGCTGTGGATACCTGTTTAG 411
Vicugna pacos                -CCTTGCCCATCG----------------GGGCCGCGGATACCTGCTTAG 450
Tarsius syrichta             NNNNNNNNNNNNN----------------NNNNNNNNNNNNNNNNNNNNN 341 ●
Myotis lucifugus             -AATCGCTC--------------------GAAATTTTGTTGTTTTCTGTG 351 ●
Pteropus vampyrus            -AATCGCCCATCGCGGTCTTTTATGCACGGAAAATTGGTTGTCATCAGTG 353 ●

                                          --->.#9.<---
Mus musculus                 A---TGGTTTT-GTGAGTTATTGCACTACCTGGA---------------- 451 ●
Rattus norvegicus            AA--TGGTTTT-GTGAGTTATTGAACT----------------------- 432 ●
Ellobius lutescens           ATGTTGGTTTT-GTGAGTTATTGCACTGCCTGGAATATCCATAACTTTTT 466 ●
Equus caballus               TTGTTGGTTTTTGTGGTTCGTTGTACTATCTGGA---------------- 403 ●
Pan troglodytes              ATTTTTTTTTT--TCATCGCCCATCGGTGCTTTTTATGGATGAAAAAATG 426 ●
Gorilla gorilla              ATTTTTTTTTT--TCATCGCCCATCGGTGCTTTTTATGGATGAAAAAATG 425 ●
Pongo pygmaeus               ATTTTTTTT----TCATCGCCCATCGGTGCTTTTTATGGATGAAAAAATG 424 ●
Homo sapiens                 ATTTTTTTTTT--TCATCGCCCATCGGTGCTTTTTATGGATGAAAAAATG 422 ●
Macaca mulatta               ATTTTTTTTT---TCATCGCCCGTCGGTGCTTTTTATGGATGAAAAAATG 423 ●
Callithrix jacchus           ATTTT--------------------------------------------- 376 ●
Echinops telfairi            ATTCTTGTTTT--------------------------------------- 420 ●
Cavia porcellus              ACTTCTTTGAT--TTCTTG----TAGAAGCTCTTTAT------------- 369 ●
Tursiops truncatus           ATTTTTTTTTT--TCATCGCCCATCGGGGCTTTTTATGGATGGAAAAGTG 385 ●
Oryctolagus cuniculus        ATTTTTTTTTT--CCATCGCCCATCGGGGCCTTTCATGGATGAAAATGTG 449 ●
Erinaceus europaeus          ATTTTTTTTCC--ATCGTCCA----------------------------- 419 ●
Sorex araneus                --------------------------------------------------     ●
Felis catus                  ATT----------------------------------------------- 403 ●
Bos taurus                   ATTTTTGTTTT--ACACCACCCATCGGGGCTTTATATGGTTGGAAAAGTG 468 ●
Sus scrofa                   ATTTTTTTTT---------------------------------------- 399 ●
Tupaia belangeri             ATTTTTGTTTT--TATCTCAATCGCTCATCGGGGCTTTTTATGGATGAAA 483 ●
Microcebus murinus           ATTTTTTTTTT--CCATCGCCCATCGGGGCTTCCCATGGATGAAAGGCGT 434 ●
Canis lupus familiaris       ATTTTTTTTTT-CTCATTGCCCATCGGGGCCTTTTATGGATGGAAGTGTT 432 ●
Ailuropoda melanoleuca       ATTTTTTTTTTTCTCATTGCCCATCGGGGCCTTTTATGGATGGAAGTGTT 461 ●
Vicugna pacos                ATTTTTTTTTTCATCGCCCATCGGGGCTTTTTGATGGATGGAGAAGTGTT 500 ●
Tarsius syrichta             NNNNNNNNNNNNNNNNNNN------------------------------- 360 ●
Myotis lucifugus             GTTCGTTATACTATCTGGAATGTCTACAAATTTTTGCTGCTAATCTTTGG 401 ●
Pteropus vampyrus            GTTCGTTGTACTATCTGATATGTCT----CTTTTCGCCGTTAA------- 392 ●
```

B

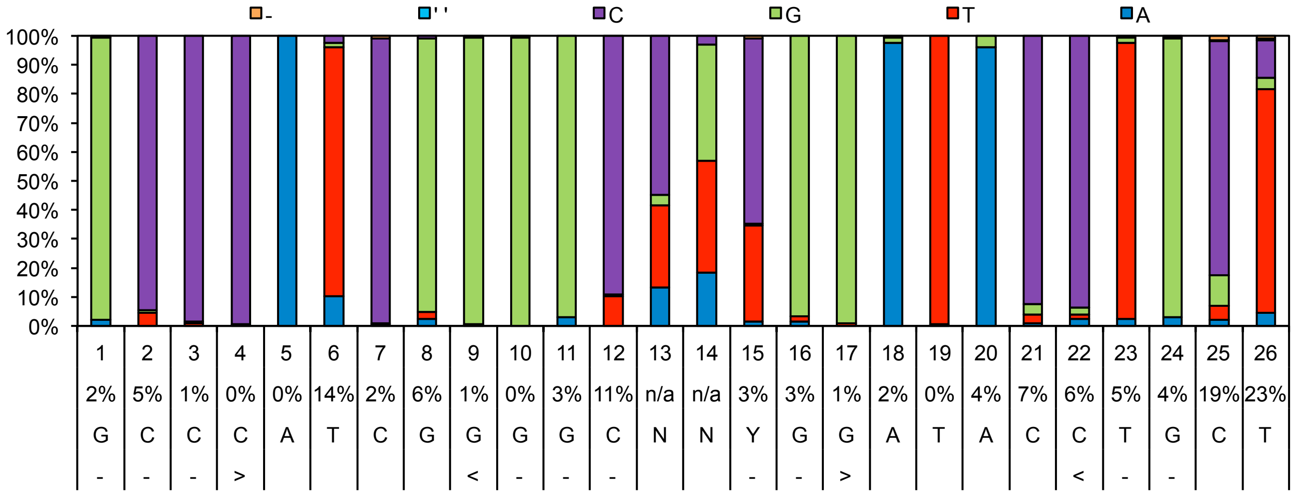

Figure S2. Alignment of repeat A sequences in 27 mammals.
(A) Sequence alignment of repeat A region in 27 mammalian species. Black circles mark sequences that were not considered *bona fide* repeat A units and were thus excluded from further analyses.
(B) Sequence conservation of 202 core repeat A units among 27 mammalian species. Lines on the X axis depict (from top to bottom) position of bases, percent of units that deviate from canonical sequence, the canonical sequence and arrows corresponding to bases forming the hypothesized stem 1 and stem 2.
