# Supplementary material for: XIST-induced silencing of flanking genes is achieved by additive action of repeat a monomers in human somatic cells
Source: Epigenetics Chromatin. 2013 Aug 1;6:23. doi: 10.1186/1756-8935-6-23 (PMC3734131; doi:10.1186/1756-8935-6-23)
Supplement: Additional file 3: Figure S3 — In silico prediction of repeat A mutant structure. Structures and free energies of 2-mer repeat A and mutants created to enforce pairing within each monomer (A1, A2) or between the two monomers (B1, B2) predicted by mfold. Bases diverging from the canonical repeat A sequence are capitalized and highlighted. [file 1756-8935-6-23-S3.pdf]

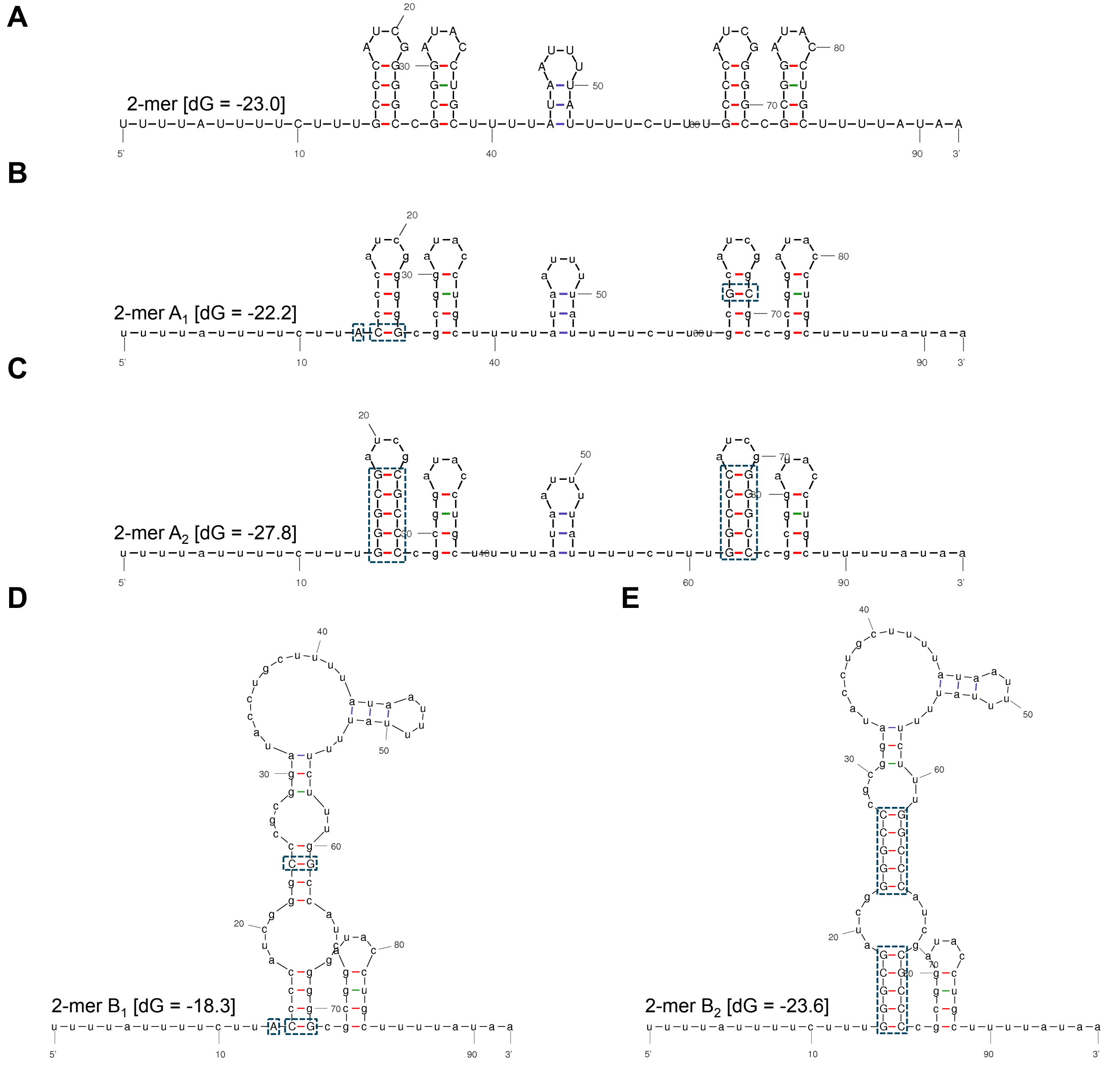

Figure S3. *In silico* prediction of repeat A mutant structure.
Structures and free energies of 2-mer repeat A and mutants created to enforce pairing within each monomer (A1, A2) or between the two monomers (B1, B2) predicted by mfold. Bases diverging from the canonical repeat A sequence are capitalized and highlighted.
