# Supplementary material for: XIST-induced silencing of flanking genes is achieved by additive action of repeat a monomers in human somatic cells
Source: Epigenetics Chromatin. 2013 Aug 1;6:23. doi: 10.1186/1756-8935-6-23 (PMC3734131; doi:10.1186/1756-8935-6-23)
Supplement: Additional file 4: Figure S4 — Analysis of repeat A sequences in 27 mammals. (A) Illustration of the approach used to analyze repeat A sequence alignment data in Figure 5A, B and Additional file 3: Figure S3B, C. (B) Analysis of reciprocal mutations in the stem 1 of individual repeat A units. The table depicts the number of occurrences when mutation in a repeat A unit would allow pairing due to the existence of a reciprocal mutation within the same unit (highlighted), in a different unit or when no reciprocal mutation exists in the species’ repeat A (listed in the last column). (C) As in B), but stem 2 is analyzed. [file 1756-8935-6-23-S4.pdf]

A

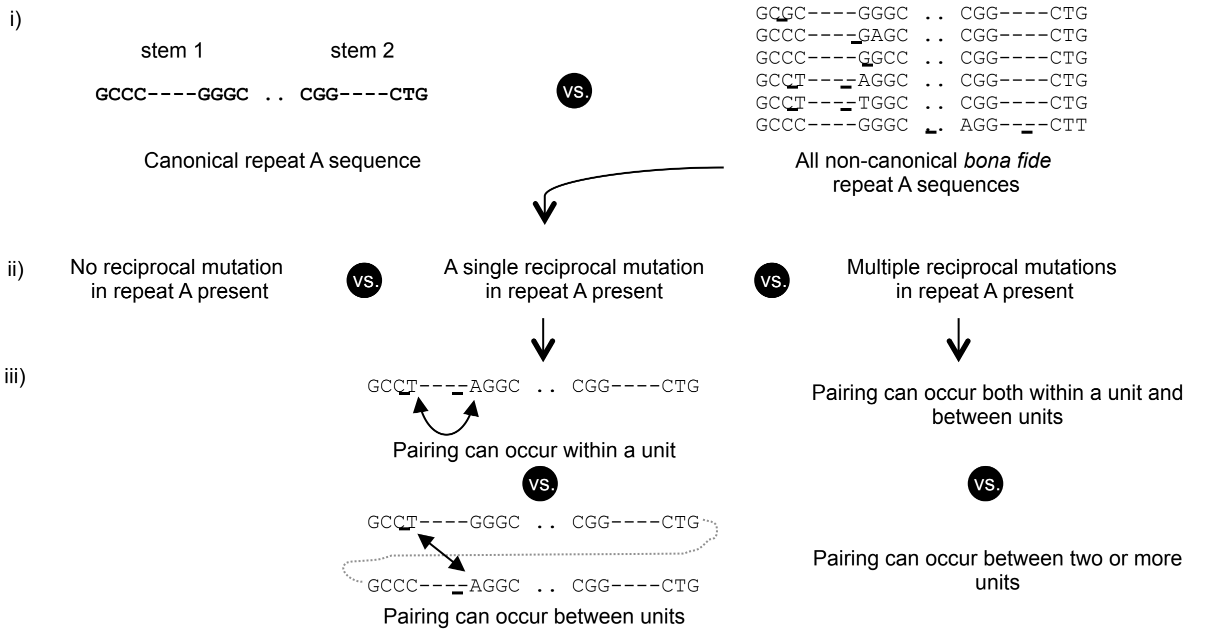

B

**Stem 1**		Creates pair with unit								
		1	2	3	4	5	6	7	8	-
Mutation in unit	1	0	0	0	0	0	0	0	0	2
	2	0	8	0	0	2	2	0	0	4
	3	0	1	0	0	0	1	0	0	1
	4	0	0	0	0	0	0	0	0	5
	5	0	3	0	0	10	0	0	1	10
	6	0	3	1	0	0	4	0	0	0
	7	0	0	0	0	0	0	0	0	1
	8	0	0	0	0	1	0	0	0	1

C

**Stem 2**		Creates pair with unit								
		1	2	3	4	5	6	7	8	-
Mutation in unit	1	0	2	1	1	0	0	1	0	7
	2	1	8	4	3	0	1	3	3	6
	3	0	1	0	0	0	0	0	0	0
	4	1	0	0	0	0	0	0	0	3
	5	0	0	0	0	0	0	0	1	3
	6	0	0	0	0	0	0	0	0	1
	7	0	0	0	0	0	0	0	0	1
	8	0	1	0	0	0	0	0	4	7

Figure S4. Analysis of repeat A sequences in 27 mammals.
(A) Illustration of the approach used to analyze repeat A sequence alignment data in Fig. 5A, B and Fig. S2B, C.
(B) Analysis of reciprocal mutations in the stem 1 of individual repeat A units. The table depicts the number of occurrences when mutation in a repeat A unit would allow pairing due to the existence of a reciprocal mutation within the same unit (highlighted), in a different unit or when no reciprocal mutation exists in the species' repeat A (listed in the last column).
(C) As in B), but stem 2 is analyzed.
