# Supplementary material for: XIST-induced silencing of flanking genes is achieved by additive action of repeat a monomers in human somatic cells
Source: Epigenetics Chromatin. 2013 Aug 1;6:23. doi: 10.1186/1756-8935-6-23 (PMC3734131; doi:10.1186/1756-8935-6-23)
Supplement: Additional file 5 — Supplementary methods. List of accession numbers or sequence coordinates of repeat A sequences used in sequence analyses and table of primer sequences and ChIP methods. [file 1756-8935-6-23-S5.pdf]

Supplementary Methods

List of GenBank accession numbers or sequence coordinates of repeat A sequences used in sequence analyses

Species	Accession number / genomic location
Mus musculus	NR_001463
Rattus norvegicus	chrX:91,467,666-91,468,097 Nov. 2004
Ellobius lutescens	EU086094.1
Equus caballus	U50911.1
Pan troglodytes	chrX:73,645,114-73,645,544 Oct. 2010 assembly
Gorilla gorilla	chrX:71,018,187-71,018,664 May 2011 assembly
Pongo pygmaeus	chrX:71,294,280-71,294,715 Jul. 2007 assembly
Homo sapiens	NR_001564
Macaca mulatta	chrX:72,974,560-72,974,994 Jan. 2006 assembly
Callithrix jacchus	chrX:65,411,057-65,411,432 Mar. 2009 assembly
Echinops telfairi	scaffold_298824:9,733-10,152 Jul. 2005 assembly
Cavia porcellus	scaffold_26:23,393,897-23,394,265 Feb. 2008 assembly
Tursiops truncatus	scaffold_92440:418-831 Jul. 2008 assembly
Oryctolagus cuniculus	U50910.1
Erinaceus europaeus	scaffold_354641:1,200-1,618 Jun. 2006 assembly
Sorex araneus	scaffold_229162:51,879-52,334 Oct. 2005 assembly
Felis catus	chrUn_ACBE01438274:3,390-3,792 Dec. 2008 assembly
Bos taurus	NR_001464.2
Sus scrofa	CU855548.6
Tupaia belangeri	scaffold_148376:1,812-2,307 Jun. 2006 assembly
Microcebus murinus	scaffold_20625:5,197-5,658 Jun. 2007 assembly
Canis lupus familiaris	chrX:60,410,297-60,410,751 May 2005 assembly
Ailuropoda melanoleuca	GL194824.1:76,507-76,993 Dec. 2009 assembly
Vicugna pacos	scaffold_25540:1,149-1,688 Jul. 2008 assembly
Tarsius syrichta	scaffold_135455:2,195-2,478 Aug. 2008 assembly
Myotis lucifugus	GL429771:10,780,415-10,780,834 Jul. 2010 assembly
Pteropus vampyrus	scaffold_7187:74,526-74,917 Jul. 2008 assembly

List of PCR primers:

Primer name	Sequence	Notes
qXIST_-1kb F	CTGCTCTGATGCCGCATAGTT	p1 in Fig. 1A
qXIST_-1kb R	TTTTGCTCGCGCACTACTCA	
qXIST 5 F	TCAGCCCATCAGTCCAAGATC	p2 in Fig. 1A
qXIST 5 R	CCTAGTTCAGGCCTGCTTTTCAT	
qpFRT_4719 F	GCTCAGAAGAAATGCCATCTAGTG	p3 in Fig. 1A
qpFRT_4790 R	TTTTTTGGAGGAGTAGAATGTTGAGA	
qpFRT_5921 F	CCACCAACAGCAAAAAAATGAA	p4 in Fig. 1A
qpFRT_5986 R	ACTCATGAAAATGGTGCTGGAA	
qpcDNA5 F3	CGCCATCCACGCTGTTTT	qRT-PCR of XIST expression, p5 in Fig. 1A
qpcDNA5 R3	CCGGAGGCTGGATCGGT	
GFPc F	ACTACAACAGCCACAACGTCTATATCA	qRT-PCR
GFPc R	GGCGGATCTTGAAGTTCACC	
qEGFP594 F	AGCGCTACCGGACTCAGAT	
qEGFP649 R	GTACCGTCGACTGCAGAATTC	
qACTB 1	TTGCCGACAGGATGCAGAA	
qACTB 2	GCCGATCCACACGGAGTACTT	
qHyg F	CAGCGAGAGCCTGACCTATTG	
qHyg R	CAGGCAGGTCTTGCAACGT	
qHyg P2 F	CGCCCCATGGCTGACTAAT	
qHyg P2 R	CAAAAAAGCCTCCTCACTACTTCTG	
qEGFP594 F	AGCGCTACCGGACTCAGAT	qPCR - ChIP
qEGFP649 R	GTACCGTCGACTGCAGAATTC	
qPgk1 1F	GGCACTTGGCGCTACACAA	
qPgk1 1R	CCTACCGGTGGATGTGGAAT	
qPgk1 3F	AGCGGCCAATAGCAGCTTT	
qPgk1 3R	CCCCTTCCCAGCCTCTGA	
qPgk1 4F	TCTGCCGCGCTGTTCTC	
qPgk1 4R	GATGGATGCAGGTCGAAAGG	
qMYT1 F	GCTACAGCAGCTACCAGGGAAT	
qMYT1 R	CTCTTCCACCAGGGTCTCTTCA	
qAPRT F	GCCTTGACTCGCACTTTTGT	qPCR - ChIP
qAPRT R	TAGGCGCCATCGATTTTAAG	
qHyg P1 F	TCAGAGGTTTTCACCGTCATCAC	
qHyg P1 R	CACCCTAACTGACACACATTCCA	
qHyg P2 F	CGCCCCATGGCTGACTAAT	
qHyg P2 R	CAAAAAAGCCTCCTCACTACTTCTG	
qCLDN16 P1 F	CCCTGTGTGAAATGTCAGCAA	
qCLDN16 P1 R	AGAGCAAACCAATTCCAAAAGC	
qCLDN16 P2 F	CCATGAGGGAAAAGTAATAATATAGGAAA	

qCLDN16 P2 R	TTGTGCCTGTAAGTCGCTTAGAAT	
pEGFP_1296 F	CGCCCTGAGCAAAGACCCCAACGA	Inverse PCR to identify transgene integration site
pEGFP_704 R	ACAGCTCCTCGCCCTTGCTCACCA	
BCL6_SNP_F1	Biotin-GTTGGGGACTGGAGGTCAAG	Pyrosequencing primer set examining rs1056932, 56.3°for 30 secs
BCL6_SNP_R1	ATCTGACTGCCAGCCCAACTC	
BCL6_SNP_S1	TTCATCGTGCTCAACA	
LPP_SNP_F1	Biotin-CTGGCATCTCTGCAGCTCAA	Pyrosequencing primer set examining rs1064607, 56.3°for 30 secs
LPP_SNP_R1	CCACCCCCTTAATCAGCACA	
LPP_SNP_S1	CACATTGTTATTTCTGTAGG	
OPA1_SNP_F1	GAATTTTCCCGCTTTATGACAGA	Pyrosequencing primer set examining rs9851685, 58.3°for 30 secs
OPA1_SNP_R1	Biotin-CGCAAAGTCATTCCACTTGTGT	
OPA1_SNP_S1	TTTGATAAACTTAAAGAGGC	
SKIL_SNP_F1	Biotin-ATTTGGTTGCATCATTTTGACTT	Pyrosequencing primer set examining rs3772172, 56.3°for 30 secs
SKIL_SNP_R1	AAAAGTTTATGCCATTCCTTGTG	
SKIL_SNP_S1	TTCCTTGTGCCATAAA	
TTC14_SNP_F1	Biotin-TTCCGCTTCCTGTACCACCC	Pyrosequencing primer set examining rs1532485, 58.3°for 30 secs
TTC14_SNP_R1	TAGTTCCCTGTTCCGTGTCCG	
TTC14_SNP_S1	CGTGTCCGCTACTTGA	

Chromatin immunoprecipitation

All steps were performed as published previously (Leung et al., 2011); incubation with micrococcal nuclease for 8 minutes provided ideal size of chromatin fragments. Antibodies used were: 5 μg of anti-H3K27me3 (07-449; Millipore), 7.5 μg of anti-H3 (H9289; Sigma), 10 μg of IgG (I8140; Sigma).

Leung, D. C., K. B. Dong, I. A. Maksakova, P. Goyal, R. Appanah, S. Lee, M. Tachibana, Y. Shinkai, B. Lehnertz, D. L. Mager, F. Rossi, and M. C. Lorincz. 2011. Lysine methyltransferase G9a is required for de novo DNA methylation and the establishment, but not the maintenance, of proviral silencing. Proc Natl Acad Sci U S A **108:**5718-23.
